# Light‐Driven, Dynamic Assembly of Micron‐To‐Centimeter Parts, Micromachines and Microbot Swarms

**DOI:** 10.1002/advs.202402263

**Published:** 2024-06-24

**Authors:** Konstantin Polev, Govind Paneru, Valentin Visyn, Olgierd Cybulski, Slawomir Lach, Diana V. Kolygina, Evelyn Edel, Bartosz A. Grzybowski

**Affiliations:** ^1^ Center for Algorithmic and Robotized Synthesis (CARS) Korea's Institute for Basic Science (IBS) Ulsan 44919 South Korea; ^2^ Department of Biomedical Engineering Ulsan National Institute of Science and Technology (UNIST) Ulsan 44919 South Korea; ^3^ Department of Physics Ulsan National Institute of Science and Technology (UNIST) Ulsan 44919 South Korea; ^4^ Department of Chemistry Ulsan National Institute of Science and Technology (UNIST) Ulsan 44919 South Korea

**Keywords:** dynamic assembly, functional architectures, light, swarms

## Abstract

This work describes light‐driven assembly of dynamic formations and functional particle swarms controlled by appropriately programmed light patterns. The system capitalizes on the use of a fluidic bed whose low thermal conductivity assures that light‐generated heat remains “localized” and sets strong convective flows in the immediate vicinity of the particles being irradiated. In this way, even low‐power laser light or light from a desktop slide projector can be used to organize dynamic formations of objects spanning four orders of magnitude in size (from microns to centimeters) and over nine orders of magnitude in terms of mass. These dynamic assemblies include open‐lattice structures with individual particles performing intricate translational and/or rotational motions, density‐gradient particle arrays, nested architectures of mechanical components (e.g., planetary gears), or swarms of light‐actuated microbots controlling assembly of other objects.

## Graphical abstract

Particles sized from microns to centimeters and placed on a fluidic bed characterized by low thermal conductivity can be manipulated and controlled by low power light. The image shows complex “planetary” motions of an ensemble of 12 parts: The rotary motions of the orbits and of the individual particles are indicated by curved, dashed arrows.

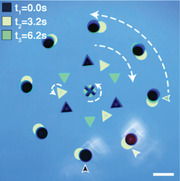

## 1 Introduction

Dynamic assembly driven by uniform or spatially modulated light irradiation has been pursued vigorously for almost two decades^[^ ^1^ ^]^ to assemble macromolecules,^[^ ^2^ ^]^ nanoparticles,^[^ ^3^ ^]^ and colloids^[^ ^4^ ^]^ into a range of non‐equilibrium structures, swarms^[^ ^5^ ^]^ and functional materials (e.g., switchable catalysts,^[^ ^6^ ^]^ nano‐ and micromotors,^[^ ^7^ ^]^ encryption systems,^[^ ^8^ ^]^ and more). Light‐based techniques to address individual objects and to manipulate and/or assemble them into dynamic formations have been developed for even longer – in particular, different types of optical tweezers^[^ ^9^ ^]^ have proven of immense value in manipulating atoms, micro‐ and nanoscale particles, or biological specimens. Still, at realistic light intensities, all these techniques impart forces too small in magnitude^[^ ^10^ ^]^ to address larger entities such as parts of MEMS,^[^ ^11^ ^]^ micromachines/microrobots,^[^ ^12^ ^]^ or watches.^[^ ^13^ ^]^ For such larger objects, light would be an appealing modality of assembly as it could circumvent the problems of wear‐and‐tear, friction, or tribocharging associated with conventional mechanical micromanipulation.^[^ ^14^ ^]^

Confronted with such limitations, various techniques combining light with other energy fields have been proposed.^[^ ^15^ ^]^ Of these, optothermal methods – merging optical forces with localized thermal effects to create tailored temperature gradients around target objects – garnered much recent interest^[^ ^15e^ ^]^ enabling finely tuned, feedback‐controlled assembly of colloidal particles in bulk liquids.^[^ ^16^ ^]^ Light manipulation at liquid‐liquid and liquid‐air interfaces has been another longstanding approach with the premise that such interfaces offer reduced drag and, at smaller scales, can benefit from capillary phenomena to support objects denser than the fluid. These interfacial systems^[^ ^17^, ^18^ ^]^ typically rely on thermocapillary effects whereby laser light heats the particles and the surrounding fluid locally, generates a gradient of temperature and of surface tension, induces Marangoni flows, and ultimately moves the target object(s). However, most of these 2D arrangements allow for only marginal control of motion,^[^ ^17^, ^19^ ^]^ operate on timescales of minutes (effective speeds of controllable motion ≈1–100 µm s^−1^;^[^ ^17^, ^20^ ^]^), or require high power densities (∼kW cm^−2^;^[^ ^17^, ^19^, ^20^ ^]^). Systems based on direct heating of the liquid phase (e.g., water heated locally by a 1455 nm laser matching water's peak absorption,^[^ ^21^ ^]^) can operate at lower light intensities and achieve higher particle speeds but, to date, have been hampered by limited resolution (determined by ∼mm‐wide size of collimated Infrared (IR) beam) and the ability to multiplex the beam (five spots/tracked particles in ref. [21]).

All in all, the existing systems are not yet light‐driven “assembly lines” that would work in a programmable manner, combining speed with precision, and assembling large numbers of objects, including those of different sizes and/or shapes. Here, we describe such an assembly platform, in which irradiation with visible light can control intricate motion patterns and dynamic assembly protocols of up to hundreds of particles differing is size by unprecedented four orders of magnitude (and in mass, by over billion of times). Our approach rests on the use of a fluidic bed characterized by low thermal conductivity assuring that light‐generated heat remains localized and sets strong convective flows in the immediate vicinity of the particles being targeted (either directly or via the irradiation of the surrounding fluid). This, in turn, translates into large magnitudes of forces elicited by even weak irradiation, allowing the system to work in two modalities: i) under closed‐loop guidance of an image recognition algorithm controlling a low‐power (<10 mW and usually <1 mW per particle) laser beam or ii) under light‐patterns from an ordinary slide projector. With these modalities, we demonstrate a wealth of dynamic assemblies in which particles can move rapidly (mm s^−1^) yet controllably. These assemblies include open‐lattice structures with individual particles performing intricate translational and/or rotational motions, nested architectures of mechanical components (e.g., planetary gears), or swarms of light‐actuated microbots controlling assembly of other objects (e.g., single droplets and droplet crystals). This broad repertoire of suitable materials (polymers, glass, metals) and applicability to either optically opaque or transparent parts across multiple length scales suggest that this approach may open new vistas for micro‐ and macro‐assembly, especially given that the light sources are inexpensive and the fluids (fluorinert, silicone oil) are non‐hazardous, chemically inert and readily available in wholesale quantities.

## 2 Results

### 2.1 Experimental Arrangement

The objects to be assembled and manipulated range from 1.43 µm spherical colloids, to ca. 100 µm glass beads, to sub‐mm and mm‐sized PDMS (polydimethylsiloxane) plates or “gears” fabricated by micromolding,^[^ ^22^ ^]^ to mm‐ to cm‐sized brass and plastic (3D‐printed) gears. These parts are either colored and absorbing in the visible (e.g., PDMS dyed by addition of mesoporous carbon) or, as described later in the text, transparent. They are placed at an interface between liquid and air or between two liquids. In the absence of irradiation, the parts interact via capillary forces and form irregularly shaped aggregates.

**Figure 1:**
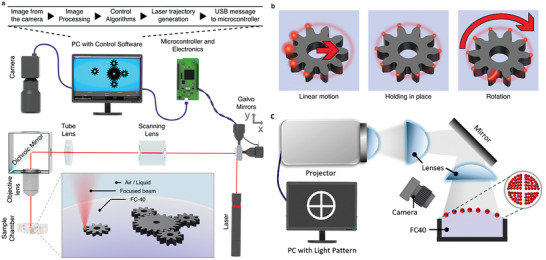
Experimental arrangements for laser‐ or projector‐controlled assembly. a) The system operates in continuous feedback whereby the software identifies particles present at the interface (within a small 6–13 mm internal diameter cuvette, see Section S1, Supporting Information), controls the galvoscanner to position the laser beam at the particles’ specific edges (to affect their motions), identifies the particles’ updated positions, and repeats the cycle to ultimately achieve a desired motion pattern. b) Schemes illustrating the principle of laser‐controlled manipulation, here of one gear‐shaped particle. Laser beam trajectories are shown as red glow, regions of beam intersection with the gear are highlighted with orange glow. (*left*) To exercise linear motion, laser intersects the particle on the side opposite to the desired direction of motion. (*middle*) To hold the particle in place, points around its entire outer perimeter are illuminated. (*right*) When an edge or edges of individual teeth are also illuminated, the gear starts rotating; at the same time, continued illumination around the perimeter prevents the gear from moving translationally (akin to the “hold” position). Depending on which of the two edges on each tooth are illuminated, the gear can be rotated clockwise or counterclockwise. c) Scheme of the system in which particles resting at the interface are illuminated by white‐light patterns (or animations) from a desktop slide projector. In this variant, particles move away from the illuminated to the dark regions and assemble in the latter (see main text for details).

We implement two types of light control. The first (**Figure** **1**a) uses a continuous‐wave laser (either 405 nm or 647 nm) delivering ≈0.5–10 mW of power (adjusted by neutral density filters) with peak intensity ≈1 W cm^−2^. The beam is deflected in two dimensions by the Thorlabs galvoscanner (cat. # GVS002 or LSKGG4) mirrors and focused on the fluid surface by Olympus MPLN5x or MPLN20x micro‐objectives through Thorlabs CLS‐SL scanning lens and a 200 mm tube lens. The beam moves with the average speed of ≈400 mm s^−1^ when it traces a particle and ≈6000 mm s^−1^ when it travels between particles. The galvoscanner‐controlled light beam acts akin to a robotic arm of traditional assembly lines – it tracks the particles, engages them, and moves to desired locations. In the basic arrangement (Figures 1b and 3a), the beam is targeted at a particle's edge “opposite” to the desired direction of motion, and the motion itself results from a locally generated convective flows in the liquid. Later in the text (Figure 7), we also describe a system in which the particles are optically transparent but the laser heats a dye‐colored liquid surrounding them. In both scenarios, the particle's instantaneous position is captured by a camera and is fed back (up to 30 frames per second) into the house‐written software that controls the galvoscanner's mirrors and thus the beam's position and the particle's future motions. When a particle is initially detected in the image, it is assigned to a separate controller within the software and the Kalman filter^[^ ^23^ ^]^ is initialized for it. The filter's prediction is used for tracking the particle between consecutive frames (using the so‐called Hungarian method for multiple objects’ assignment^[^ ^24^ ^]^), as well as for the motion correction if the particle escapes the planned trajectory (see Section S2, Supporting Information for details on the use of Kalman filter). When many particles are present, a state‐machine‐based control planner sets the motion plan for all object controllers, manages their outputs, and converts them into an output trajectory for the laser. This laser trajectory is optimized by solving the Travelling Salesman Problem using Google or‐tools library^[^ ^25^ ^]^ (for further technical details, see Section S2, Supporting Information).

The second modality (Figure 1c) uses broad‐spectrum, incoherent light from a common liquid‐crystal display (LCD), display, projector (here, Epson EH‐TW6700W, 1.1 W cm^−2^ on a sample plane). A desired light pattern is encoded in a PowerPoint presentation and projected onto the interface. Convective flows are set up only in the irradiated regions causing the particles to move toward dark regions (or regions of lower light intensity). Unlike the “robotized” laser setup, this modality is not as flexible in terms of controlling individual particles but is technically straightforward and allows for the control of large‐area patterns.

### 2.2 Parameters of the Liquid Bed

**Figure 2:**
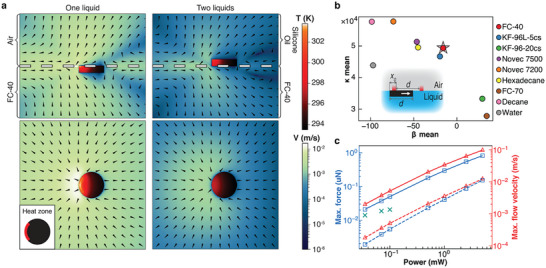
Quantification of thermocapillary flows and light‐imparted forces acting on sub‐mm particles. a) Numerical simulations quantifying particle's temperature (“red” color scale) and fluid velocity (“blue” color scale) distributions. Arrows give flow directions. Here, the particle is made of PDMS (diameter 300 µm, height 80 µm as in experiments illustrated in Figure 4) and the region on its top surface (colored red in the inset at the bottom left) is receiving 0.5 mW of heat to simulate light absorption. Top row = side views; bottom row = top views. In the left column, the particle is floating on FC‐40 and is covered by KF‐96L‐5cs silicon oil; in the right column, it is immersed in FC‐40 save for its top face that is exposed to air. Note that for the FC‐40/KF‐96L‐5 liquid/liquid interface, the thermocapillary flows are more localized to the “heated” side of the particle; when multiple particles are manipulated, they can be pushed into contact more easily (cf. Figure 4b; Movie S2, Supporting Information) than for the liquid/air interface. b) Simulations of “controllability” of particle motions between two hypothetical heat sources for different liquids (inset: the “heaters” are in red and are spaced by particle width *d*; *x_0_* is the particle's displacement at the start of a simulation). The particle is pushed to the right when its left edge is heated by the left heater, and to the left when its right edge is heated by the right heater. Ideally, for controllable motion, the particle should settle at the midpoint as rapidly as possible, akin to a dampened oscillator. The x‐axis in the plot quantifies damping by parameter β, related to viscosity, thermal diffusivity and magnitude of heating (β is highly negative when the particle tends to escape the trap; when it is highly positive, particle settles in the middle without oscillations; see also Figure S6,). The y‐axis is the elasticity parameter, κ, which depends more on the heating power (see Figure S6c, Supporting Information) and measures particle's motion speed and acceleration. Liquids like Novec 7200 or decane give rapid (high κ) but jiggly (low β) back‐and‐forth motions; liquids like viscous FC‐70 or silicone oil KF‐96‐20cs support steady (high β) but slow (low κ) motions. FC‐40 followed by silicone oil KF‐96L‐5cs provide the best tradeoff between these two regimes. The values of β and κ shown are averages of 25 simulations, starting from five different initial particle displacements *x_0_* and using five heating powers for each liquid. For further discussion and modeling details, see Section S3 (Supporting Information). c) The maximal force magnitude (*blue*) and the maximal flow velocity (*red*) dependence on heater power in 3D simulations (polygonal markers) and as measured experimentally (cross markers). Solid line is for the FC‐40/air interface, dashed line is for the FC‐40/Silicone oil interface (particle dimensions are as in panel (a)). Teal crosses are forces measured experimentally in FC‐40/air system; due to the measurement limitations, the values are available only for low laser powers (see Section S4, Supporting Information for further discussion). For the dependence of force on particle diameter, see Section S3 (Supporting Information).

In both systems, the speeds and controllability of particle motions depend crucially on the nature of the liquid(s) used. For instance, for organic solvents capable of supporting particles on the surface, for example, hexadecane, the motions are highly uncontrollable whereas for water, controllable motion is achievable only with significantly higher laser powers. Surveying multiple liquids, we established that one key factor is liquid's low thermal conductivity (see Table S2, Supporting Information) such that the laser‐generated heat remains “localized” and sets strong convective flows in the particle's immediate environment. For very small, µm‐sized colloids, stable motions without sudden “jiggles” require higher viscosity – for these particles, our liquid of choice was FC‐70 Fluorinert (thermal conductivity 0.071 W m^−1^ K^−1^, viscosity 0.024 Pa·s). For larger sub‐mm or mm particles, achieving rapid yet controllable motion requires lower (but not too low) viscosity and FC‐40 (0.067 W m^−1^ K^−1^, 0.0041 Pa·s) or, to a lesser extent, KF‐96L‐5cs silicone oil (0.12 W m^−1^ K^−1^, 0.00458 Pa·s) are typically used. Both simulations and experiments (**Figure** **2**; Sections S3 and S4, Supporting Information) indicate that for such larger particles, the light‐induced thermocapillary flows impart 10^−8^–10^−6^ N forces and mm s^−1^ speeds; these forces dominate forces due to buoyancy‐driven convection by ca. two orders of magnitude. Of note, both FC‐70 and FC‐40 are also appealing because they are relatively inexpensive, chemically inert, and non‐toxic. Of note, such strong forces results from a very small increase in the temperature of the irradiated particles (e.g., ≈1 deg in Figure S10c,d, Supporting Information).

### 2.3 Dynamic Arrays of µm to cm Particles

**Figure 3:**
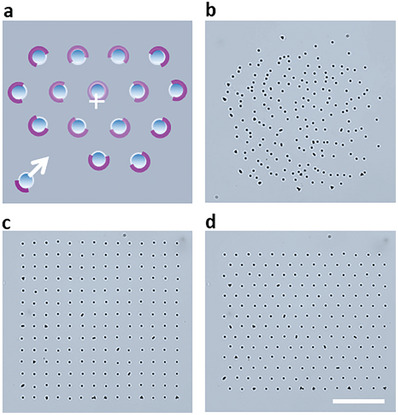
Automated dynamic assembly of micron‐sized particles using low‐power laser. a) Illustration of the control principle used to assembly the particles. Purple lines represent the arcs traced by the laser; the more intense the purple color, the slower the laser moves and, consequently, the more heat is delivered to the particles from that direction. Laser speeds are also slower in the vicinity of particles that are closer to the periphery of the formation (measured from the formation's center of mass, denoted by white “+”), or those further away from their destination points. White arrow indicates the movement direction of the last bead being assembled while other, already assembled beads, are held stationary by the beam scanning over them. b) Optical image showing random distribution of ≈200 iron‐oxide‐coated polystyrene beads with 1.43 µm diameters pinned at the FC‐70/C16 interface. Programmed ≈0.5 mW laser‐beam pattern imposed from below the dish organizes these particles first into c) a square lattice and then d) into a hexagonal lattice. Scale bar is 60 µm and same for panels (b–d). See also Movie S1 (Supporting Information).

**Figure 4:**
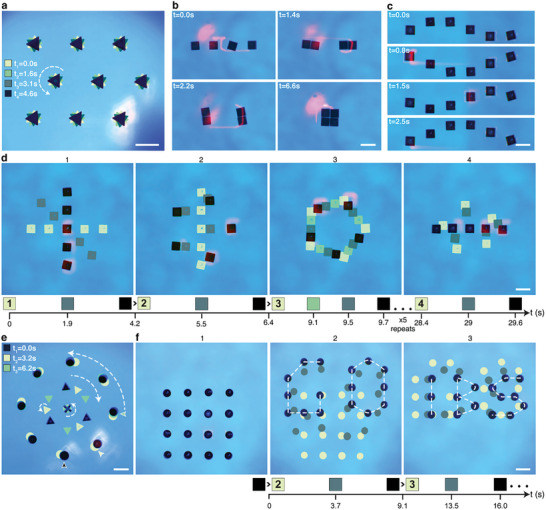
Parallelized, dynamic assembly of submillimeter parts controlled by low‐power light. Images track the motions of variously shaped PDMS particles at different instants of time (for live motions, see accompanying Movie S2, Supporting Information). All particles are floating at the FC‐40/decane interface and are simultaneously controlled by one, low‐power laser beam. a) Nine triangles arranged into a hexagonal lattice spin around their axes. b) Assembly of squares first into a pair of rectangles and then, after each rectangle is rotated, into one larger square. c) Wave‐like motion of six squares arranged into a linear structure. d) Interconversions and rotations of linear and polygonal formations of five square particles. Legends under the images show the times for each color‐coded pattern; numbers in the squares represent starting positions in images with matching numbers; arrows between squares indicate the same position between consecutive images. e) Complex “planetary” motions of an ensemble of 12 parts. The rotary motions of the orbits and of the individual particles are indicated by curved, dashed arrows. Positions of the outer‐orbit disks at t_1_ = 0 s, t_2_ = 0.04 s, and t_3_ = 0.06 s are marked by small colored arrows. f) 16 disks are first assembled into a square grid and then into patterns tracing words “GO” and “IBS”; these words are periodically interchanging. Scale bars in all images are 500 µm.

With these preliminaries, we first discuss various types of dynamic assemblies controlled by the laser‐beam system. **Figures** **3** and **4** demonstrate fully automated, parallelized, and rapid control of multiparticle assemblies and of individual particles within these assemblies. Figure 3 and Movie S1 (Supporting Information) illustrate that the lower size limit of the particles that can be manipulated matches performance of classical optical‐tweezing systems. Here, the image‐recognition algorithm recognizes 1.43 µm colloids (polystyrene beads coated with iron oxide) that are initially distributed randomly over the interface (Figure 3b), and the 405 nm laser beam engages them by tracing arc “outlines” around each particle (Figure 3a, for algorithm details, see Section S2, Supporting Information). Directional motion is then achieved by the algorithm moving these outlines until a pre‐programmed pattern is reached (see also Figure S12, Supporting Information). In Figure 3c, this pattern is a square lattice of 196 colloids and in Figure 3d, it is re‐programmed and dynamically interconverted into a hexagonal lattice. Once a desired structure is assembled, the algorithm makes sure that these objects do not deviate from the intended trajectories and if they do –, e.g., upon mechanical disturbance of the system – automatically corrects their trajectories and re‐assembles the dynamic target pattern.

In Figure 4 and Movie S2 (Supporting Information), the 647 nm laser “arm” and control algorithm are used to manipulate particles that are ca. million times more massive than the colloids from Figure 3. This set of experiments is also intended to demonstrate precision with which individual objects within larger assembles can be addressed. Specifically, differently‐shaped parts (triangles, squares, rectangles, disks) are all made of dye‐doped PDMS, and are ca. 300 µm in size and 80 µm in height. As before, the laser beam traces “outlines” of each particle but is now not only translating these outlines but also rotating them. In this way, non‐centrosymmetric particles can be moved around while being rotated around their individual axes, ultimately giving rise to a variety of intricate motion patterns. For example, in Figure 4a, triangles are brought into a hexagonal lattice that is held stationary but each triangle is made to rotate counterclockwise. In Figure 4b, pairs of squares are first assembled into rectangles, and these rectangles are then rotated and assembled into a square (this example illustrates that particles can be brought almost into direct contact since the outwardly‐directed thermocapillary flows are strong only on particles’ heated sides, see right column of Figure 2a). In Figure 4c, the linear formation of squares performs a wavy motion, and in Figure 4d, square particles are interchangeably forming linear and polygonal assemblies which, in addition, are being rotated. Figure 4e demonstrates independent control of different particle types: the target pattern comprises a central cross‐shaped particle surrounded by a smaller “orbit” of three triangles and the outer orbit of nine disks. The central cross is made to rotate counterclockwise; the triangles form an orbit that, as a whole, processes clockwise while each triangle also rotates counterclockwise around its center; the outer orbit of disks precesses counterclockwise. Finally, in Figure 4f, an initially square lattice of disks is rearranged into “GO” and then “IBS” formations, where IBS stands for our Institution's name (Institute for Basic Science).

### 2.4 Assembly of Nested Architectures

**Figure 5:**
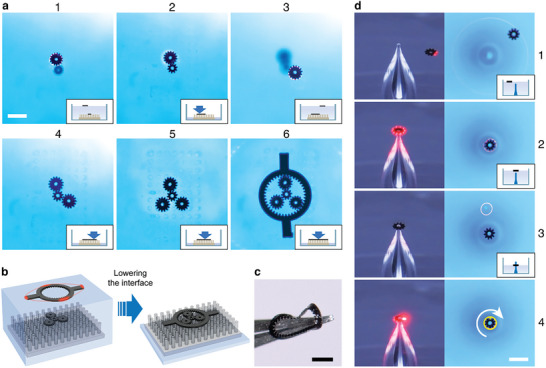
Assembly of nested architectures at two interfaces. a) Snapshots illustrating assembly of a planetary gear system. The parts are positioned and controlled by light at the top, liquid‐air interface and are combined with the already‐assembled parts at the lower solid‐liquid interface. Particles present at the lower interface are out‐of‐focus in “1” and “3” that zoom on the top interface. Other images focus on the bottom interface. See also Movie S3 (Supporting Information). b) The lower interface is microstructured with an array of 100 µm wide, 350 µm tall pillars. When a flat support was used and the parts were brought onto it upon removal of liquid, these parts experienced significant lateral capillary forces due to liquid dewetting – this adversely affected the ability to control the parts’ positions. Such problems were absent with the microstructured support. c) An optical image illustrating the sticking and deformation of a gear part manipulated by tweezers rather than by light. d) Tethering of a small gear with 95 µm inner opening onto the 80 µm shaft (made by pulling a glass capillary). Once the gear is tethered, the laser is used to rotate it around the shaft's axis. See also Movie S4 (Supporting Information). Scale bars in all images correspond to 500 µm.

Actuation confined to a planar interface is not suitable for the assembly of nested architectures such as those present in many mechanical devices. One such an architecture is the system of planetary gears which, as a proof‐of‐concept, we assembled by combining light actuation with the vertical motion of the interface (effected by removing/adding liquid from/to the cuvette) and pinning of the already‐assembled parts onto a support surface at the cuvette's bottom (see Movie S3, Supporting Information). In image “1” of **Figure** **5**a, the interface is lowered and the “sun” gear is pinned, the liquid is replenished, and the first planetary gear at the interface is positioned above the “sun”. When the liquid is removed – while the laser‐and‐image‐recognition system keeps the planetary gear in position – the two gears are engaged (image “2”). The operation is repeated for the remaining two planetary gears (“3”–“5”) and, finally, an outer gear is brought into position and lowered to complete the assembly at the pinning surface (“6” and Figure 5b). We note that achieving this assembly by mechanical means might be rather difficult given the flexibility and “stickiness” of the thin components, as illustrated in Figure 5c. Also, the precision with which the components can be manipulated and controlled is worth emphasizing – this is illustrated in Figure 5d and Movie S4 (Supporting Information), whereby a gear with 95 µm diameter of the inner opening is positioned above, lowered, and ultimately threaded onto a 80 µm‐wide glass shaft.

### 2.5 Swarms of Light‐Actuated Microbots

**Figure 6:**
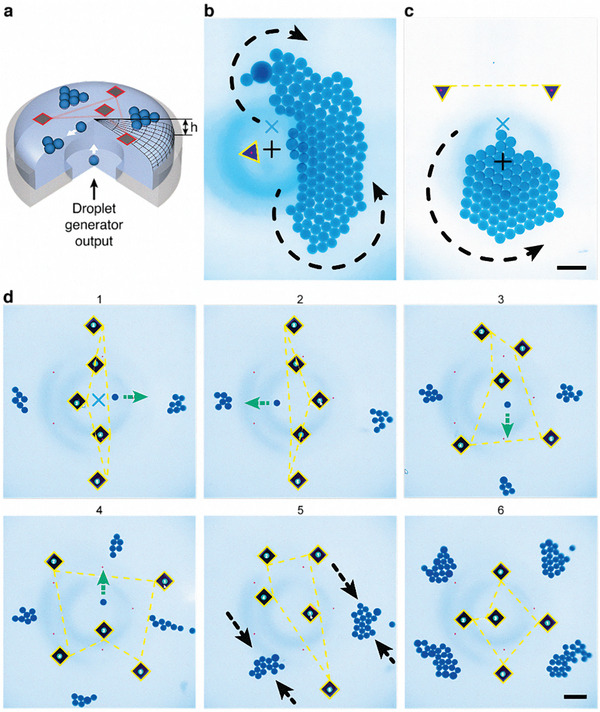
Manipulation of droplets and droplet crystals by swarms of light‐controlled microbots. a) Scheme of the experimental arrangement. A T‐junction system is embedded in the floor of the cuvette and produces dyed water droplets in FC‐40 carrier liquid. Droplet raise to the FC‐40/air interface and are manipulated by the light‐driven microbot particles. b) One triangular microbot stretches and arches a growing droplet crystal (blue “x” indicates the position where droplets emerge on the surface and black “+” indicates the center of the cuvette). c) Two triangular microbots work in unison to rotate and “shape” a droplet crystal. d) A swarm of five square‐shaped microbots is programmed to push incoming droplets to four different locations of the interface (repeating a cycle right/left/down/up indicated by the green arrows; black arrows indicate merging of two pairs of clusters). Yellow lines trace laser's trajectory. Laser moves 15 times faster between the particles (dashed lines) than when tracing outlines (solid lines) of and moving the particles. Ultimately, droplets form four smaller assemblies shown in the bottom‐right panel. For real‐time dynamics of all systems, please see Movie S5 (Supporting Information). Scale bars in (b–d) are 500 µm.

Aside from just being assembled, the light‐addressable parts can also play active roles and be used as microbots organizing other components. This capability is illustrated in **Figure** **6**a–d and Movie S5 (Supporting Information) in which dye‐colored water droplets are delivered onto the surface of FC‐40 via a T‐junction droplet generator at the bottom of the cuvette (Figure 6a). Because the interface is made convex, the lighter liquid droplets form close‐packed arrays near the cuvette's axis. When the laser‐actuated particles are also present, they can generate flows in the plane of the interface and thus exert forces on the droplets and/or droplet assemblies. In Figure 6b, one such triangular microbot is pushing against a droplet crystal making it elongated and “arched”. In Figure 6c, two microbots move and work in unison to rotate the assembling droplet crystal, in effect making it more regularly shaped. In the sub‐panels of Figure 6d, a small swarm of five laser‐controlled microbots continuously reorients to direct the incoming droplets to four different locations at the interface. Ultimately, four smaller droplet crystals are formed in these locations. We emphasize that in all of these demonstrations, the droplets being manipulated are not heated by the laser, which could be of importance if these droplets contained temperature‐sensitive or biological molecules.

### 2.6 Extensions to Transparent Parts and White‐Light Illumination

**Figure 7:**
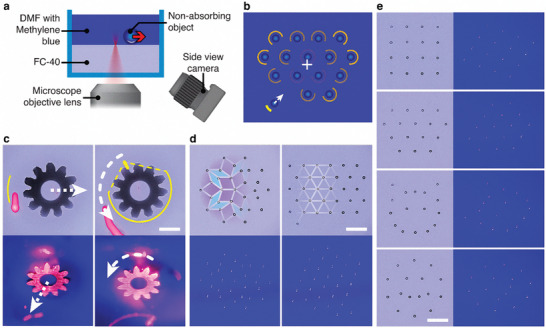
Assemblies of non‐absorptive objects. a) Scheme of the setup used for manipulation of non‐absorptive objects at the FC‐40/dyed DMF interface. Panel b) illustrates the control principle used in such experiments. Yellow lines represent the shapes traced by the laser; the more intense the yellow color, the slower the laser moves and, consequently, the more heat is delivered to the dyed liquid at a specific location. Laser speeds are slower in the vicinity of objects that are closer to the periphery of the formation (measured from the formation's center of mass, denoted by white “+”), or those further away from their destination points. White arrow indicates the movement direction of the last bead being assembled while other, already assembled particles, are held stationary. c–e) Experimental microscope (top row in (c) and (d), left column in (e)) and side‐view camera (bottom row in (c) and (d), right column in (e)) images of non‐absorptive objects manipulated/organized at the interface between FC‐40 and dyed DMF, also demonstrated in Movie S6 (Supporting Information). c) Translational motion (*left*) and rotation (*right*) of a 2.7 mm brass gear held at the interface by capillarity. d) 31 hollow glass beads, each 120–150 µm in diameter, arranged into and interconverted between quasicrystalline (Penrose P3, *left*) and hexagonal (*right*) lattices; the lattices are indicated in partial overlay. More patterns are shown in e) whereby 16 of the same glass beads form, top to bottom, square and hexagonal lattices, a cat, and a smiley face. Scale bars in (c) to (e) are 1 mm.

The method can be further extended to transparent or reflective parts by heating not these particles but the fluid surrounding them. In the introduction, we have mentioned a system of this kind based on infrared illumination causing localized water heating.^[^ ^17^, ^21^ ^]^ With the fluidic bed our systems uses, we can use visible light and achieve localized heating by coloring the liquid with a light‐absorbing dye. This is illustrated in **Figure** **7**a–e and Movie S6 (Supporting Information), whereby the upper‐phase DMF (dimethylformamide) liquid is dyed with methylene blue (the lower FC‐40 phase does not dissolve common dyes and cannot easily be dyed). The ≈1–10 mW laser beam is reaching the DMF/FC‐40 interface from the bottom, and traces arcs of various angles around the particles (Figure 7b, for algorithm details, see Section S2, Supporting Information). The thermocapillary flows induced in the optically absorptive liquid are strong enough to move and rotate a large reflective brass gear in Figure 6c, or to organize and rapidly interconvert various open‐lattice formations of transparent glass beads shown in Figure 7d,e and Movie S6 (Supporting Information).

### 2.7 Assemblies Formed Under Projector Light

**Figure 8:**
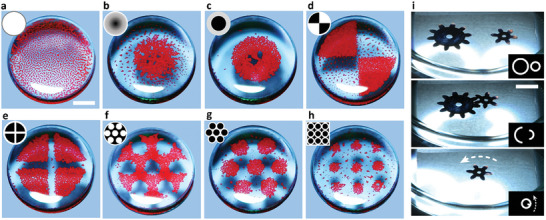
Assemblies driven by white‐light illumination. For the set‐up using the conventional slide projectors as the light source, see Figure 1c. In a–h), the FC‐40/air interface supports red‐colored, 125–150 µm polyethylene beads; in i) it supports much larger 6–10 mm gears. In all cases, these objects are assembled or manipulated by patterns of light (shown in the insets) coded in PowerPoint and projected onto the interface from the slide‐projector. Scale bars are 5 mm. See also Movie S7 (Supporting Information).

Last but not least, broad‐spectrum incoherent light from a common LCD projector (here, Epson EH‐TW6700W, 1.1 W cm^−2^ on a sample plane) can be used instead of a laser – this modality is not as flexible in terms of controlling individual particles but is technically straightforward and allows for the control of large‐area patterns. To begin with, in **Figure** **8**a, the FC‐40/air interface is covered with red‐colored polyethylene beads and is subject to uniform, white light illumination (this and other illumination patterns are illustrated in the insets). As particles absorb the incident light, they heat up, set up convective flows directed outward from each particle, and thus give rise to interparticle repulsions – as a result, the particles form an open lattice over the entire interface. By contrast, in Figure 8b, the illumination pattern is not uniform with light intensity increasing along the radial direction. The convective flows and interparticle repulsions are weakest near the “dark” center and increase toward the edges of the dish – consequently, the particles form a gradient of density, from the densest surface coverage near the center and sparsest near the periphery. Finally, in Figure 8c–h, the imposed PowerPoint light patterns are binary, that is, comprised of the white and dark regions. Due to the repulsive interparticle interactions only in the illuminated regions, the particles migrate, within few seconds, into the dark regions. As illustrated in Movie S7 (Supporting Information), the assemblies formed in this manner are stable in the presence of a given light pattern but can be re‐shaped upon imposition of another pattern. We note that LCD‐projector light patterns can also be used to manipulate much larger objects, as demonstrated by the assembly of 6 and 10 mm gears in Figure 8i. In this experiment, the PowerPoint supplies not a static light distribution but an animation that then guides the translational and/or rotational motions of the gears.

## 3 Conclusion

In summary, we showed that variety of parts spanning four orders in magnitude in size can be rapidly and precisely manipulated by low‐power light if placed onto a liquid supporting localized heating and strong thermocapillary flows. The combination of these physical phenomena with image recognition and particle control algorithms can pave the road to new, non‐invasive methods of assembly, particle sorting, or channel‐less microfluidics for droplet manipulation, the latter with potential uses in reaction screening^[^ ^26^ ^]^ based on droplet ensembles.

## 4 Experimental Section

### Fabrication of PDMS Particles

Particles were fabricated by double replica molding. Initially, molds of desired sizes and shapes were made by UV lithography in ≈80 µm‐thick SU‐8 photoresist (SU‐8 2075, MicroChem) supported by glass wafers. Positive photomasks delineating various geometric elements and gears were used and UV exposure time of 10–14 s was applied for optimal results. Soft baking was performed at 60 °C for 5 min, while post‐exposure baking, at 90 °C for 10 min. The post‐exposure‐baked wafers were developed in MicroChem SU‐8 developer for 10–20 min and cleaned periodically with isopropanol to check if excess photoresist had been removed. Sylgard 184 PDMS elastomer kit (Dow Corning) was mixed (10:1 w/w base to crosslinker), degassed, poured over the photolithographically‐patterned SU‐8/glass wafer, and cured for 12 h after additional degassing. Resulting PDMS master mold was plasma oxidized (2 min at 200 W, 40 sccm of air) and immediately transferred into a desiccator for silanization (2 h, 150 mbar air pressure) with 50 µL of 1H,1H,2H,2H‐perfluorodecyltrichlorosilane (Alfa Aesar, cat# L16584).

To produce light‐absorbing particles from a silanized mold, a batch of black‐dyed PDMS was prepared by introducing 200 mg of mesoporous carbon (Sigma–Aldrich, cat# 699 632) to fresh non‐cured PDMS mixture. The resulting black elastomer was mixed using a magnetic stirrer for ≈1 h at 300 rpm, degassed, and poured over the master mold. For the particles to be leveled and even, an excess of dyed PDMS was manually swept away from the mold under the microscope. The PDMS‐filled mold was then degassed and cured in the oven for 2 h at 65 °C and left at room temperature overnight. Cured black PDMS particles were manually extracted from the mold with tweezers under the microscope; small amount of isopropanol applied onto the particles facilitated the extraction.

### Fabrication of PDMS Micropillars

Micropillars were made by double replica molding, similar to the procedure described above. One significant difference was deposition of a ≈5 µm seed layer of SU‐8 3005 (MicroChem) that covered the whole wafer and was cross‐linked by spatially uniform UV exposure (i.e., without any photomask). Soft baking was 5 min at 95 °C, post exposure baking was at 70 °C for 1 min followed by ramping up to 95 °C at 5 °C min^−1^ rate and holding the temperature for 3 min. After developing for 3 min, the wafer was hard‐baked for 20 min in total, starting from 170 °C and ramping up to 200 °C at 5 °C min^−1^ rate. Afterward, a ≈350 µm‐thick layer of SU‐8 2150 (MicroChem) was spin‐coated atop of the seed layer. A photomask with a rectangular array of circular features (100 µm in diameter, 200 µm center‐to‐center spacing) was used during the exposure step to obtain cylindrical pillars; i‐line filter was used to avoid T‐topping on the pillars. Soft baking was performed at 70 °C for 10 min followed by 95 °C for 90 min; post‐exposure baking was at 95 °C for 30 min. A 10:1 base‐to‐crosslinker PDMS was poured onto the master mold, allowed to cure, and peeled off the master to yield a PDMS block with 350 µm‐tall, cylindrical features embossed on its surface.

### Liquids Used in Experiments

The liquids used in the experiments were: FC‐40 (cat# F9755, Sigma–Aldrich), FC70 (cat# F9880, Sigma–Aldrich), decane (cat# 457 116, Sigma–Aldrich), hexadecane (cat# H6703, Sigma–Aldrich), silicone oil 5 cS (item# KF‐96L‐5cs, Shin‐Etsu), silicone oil 20 cS (item# KF‐96‐20cs, Shin‐Etsu). Water was obtained from Millipore Milli‐Q purification system; water droplets were dyed with 1% w/w Methyl Blue (cat# M6900, Sigma–Aldrich). In the experiments with non‐absorptive objects, upper phase DMF (cat# 227 056, Sigma–Aldrich) was dyed with 0.04% w/w Methyl Blue (cat# M6900, Sigma–Aldrich).

### Other Materials

Microbeads were polystyrene coated with iron oxide nanoparticles (item# PS‐MAG‐COOH, 1.43 µm, microparticles GmbH), red polyethylene (item# REDPMS‐0.98, 125–150 µm, 10 g, Cospheric) and clear polyethylene (item# CPMS‐0.96, 850–1000 µm, 10 g, Cospheric). Brass gear was taken from the shaft of Tower Pro Micro Servo SG90 electric motor.

## Conflict of Interest

The authors declare no conflict of interest.

## Author Contributions

K.P. performed most experiments, built the main experimental setup, and programmed software and firmware. G.P., V.V., and K.P. built the projector setup and performed experiments on it. V.V. and K.P. performed and analyzed the computer simulations. G.P. assembled colloidal arrays in Figure 3. O.C., D.V.K., and S.L. contributed to initial experiments. O.C. performed CNC machining. S.L. and E.E. performed lithography and PDMS particles manufacturing. All authors contributed to the discussion of the results. B.A.G. conceived the project and supervised the research. K.P., G.P., and B.A.G. wrote the manuscript, and all authors contributed to writing methods’ description.

## Supporting information

Supporting Information

Supplemental Movie 1

Supplemental Movie 2

Supplemental Movie 3

Supplemental Movie 4

Supplemental Movie 5

Supplemental Movie 6

Supplemental Movie 7

## Data Availability

The data that support the findings of this study are available in the supplementary material of this article.
