# Supplementary material for: Light‐Driven, Dynamic Assembly of Micron‐To‐Centimeter Parts, Micromachines and Microbot Swarms
Source: Adv Sci (Weinh). 2024 Jun 24;11(32):2402263. doi: 10.1002/advs.202402263 (PMC11348064; doi:10.1002/advs.202402263)
Supplement: Supplementary file 1 — Supporting Information [file ADVS-11-2402263-s003.pdf]

ADVANCED SCIENCE
Open Access

Supporting Information

for *Adv. Sci.*, DOI 10.1002/advs.202402263

Light-Driven, Dynamic Assembly of Micron-To-Centimeter Parts, Micromachines and Microbot Swarms

*Konstantin Polev, Govind Paneru, Valentin Visyn, Olgierd Cybulski, Slawomir Lach, Diana V. Kolygina, Evelyn Edel and Bartosz A. Grzybowski**

Supplementary Information for manuscript entitled ***"Light-driven, dynamic assembly of micron-to-centimeter parts, micromachines and microbot swarms"***

Authors: Konstantin Polev, Govind Paneru, Valentin Visyn, Olgierd Cybulski, Slawomir Lach, Diana V. Kolygina, Evelyn Edel, Bartosz A. Grzybowski*

Correspondence to: nanogrzybowski@gmail.com

Contents:

Section S1. Experimental cuvettes.

Section S2. Image processing and control system.

Section S3. Simulations.

Section S4. Experimental measurement of force.

References

Captions for Movies S1 to **S7**

Section S1. Experimental cuvettes

Cuvettes were fabricated from transparent poly(methyl methacrylate) (PMMA) and polycarbonate by micro-milling with CNC router. **Figure S1** shows a typical cuvette used in our experiments, whereas **Figure S2** has the version with droplet generators supplying droplets through a hole in the middle of cuvette's floor (used in experiments in **Figure 6**, **a** to **d**). In all cases except the experiments with water droplets, a bottom plate (bolted under the cuvette in **Figure S1f**) has flat surfaces, and the top surface serves as a floor to the cuvette. To prevent evaporation of volatile liquids, the setup is optionally covered with a plate having a sealed glass window. Tight sealing of the entire assembly is achieved by using NBR or Viton O-rings placed between the plates in grooves marked in **Figure S1**, **a** and **b**. The cylindrical cavity in the center of the cuvette provides working area of diameter d (from 6 to 12 mm in different experiments) and height h (from 3 to 8 mm). The walls of the cavity have the thickness of $D - d \approx 0.2$ mm (see **Figure S1**, **a** and **d**) to facilitate fixation of the working fluid's meniscus – depending on its level, it may be convex or concave, resulting in the floating microobjects being either attracted or repelled from the edge of the meniscus. The working liquid can be supplied by stellate channels (100 μm wide) at the floor of the cavity (**Figure S1**, **b** and **e**) – which is especially suitable for smooth control of liquid level – or from the top by a side channel surrounding the upper flange (this way of filling the chamber is used when working with two immiscible liquids, with the lighter one added from the top).

In the version with droplet generators (**Figure S2**), the bottom plate houses microchannels arranged into three separate T-junctions (the numbered channels in **Figure S2a** are denoted as "c" for continuous-phase fluid – typically the same as the working liquid in the chamber, or "d" for droplet-phase fluid) that converge into the common outlet at the center point of the bottom plate –

from this point, droplets produced in T-junctions are freed to the cavity and, provided that they are lighter than the working fluid, come up to the surface.

The experiments with upper-phase DMF were conducted in a cylindrical glass cuvette (60 mm in diameter).

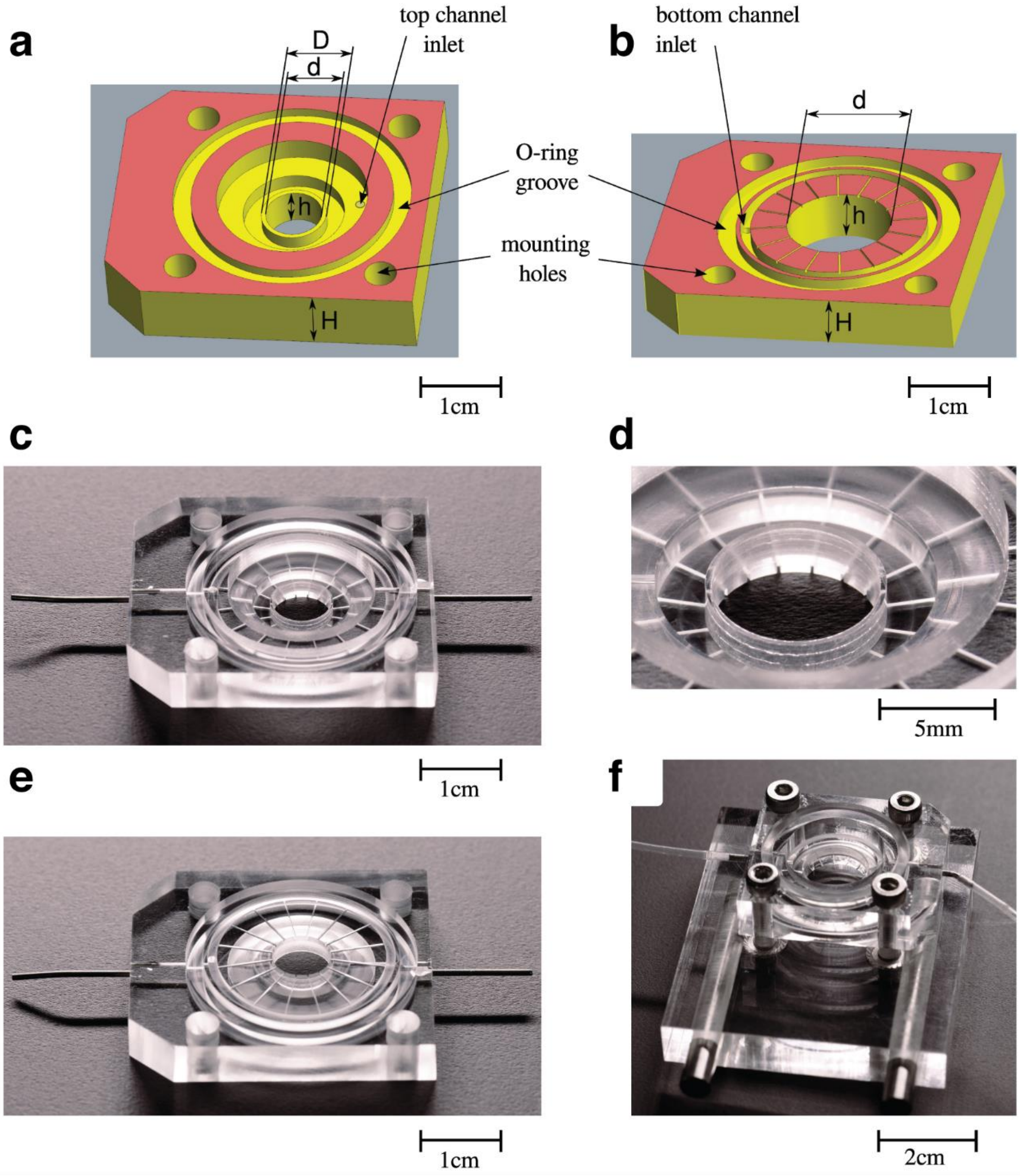

Figure S1. Cuvette used for light-driven manipulation of objects on the surface of a liquid (or at an interface between two different liquids). a,b) Simplified scheme of top and bottom sides of the chamber's main plate. **c)** Actual photograph of one such a plate, as seen from the top, and **d)** magnification of its central part with flange surrounding the workspace, used for fixing the meniscus. **e)** Back of the same plate (view from the bottom) showing stellate channels used for

filling the chamber with the working fluid and controlling its level. **f)** Top view of the mounted cuvette – the plate from panels (**a**-**e**) is attached to the bottom plate with four M4 bolts, with NBR O-ring in between. Polyethylene tubings are connected to both inlet ports.

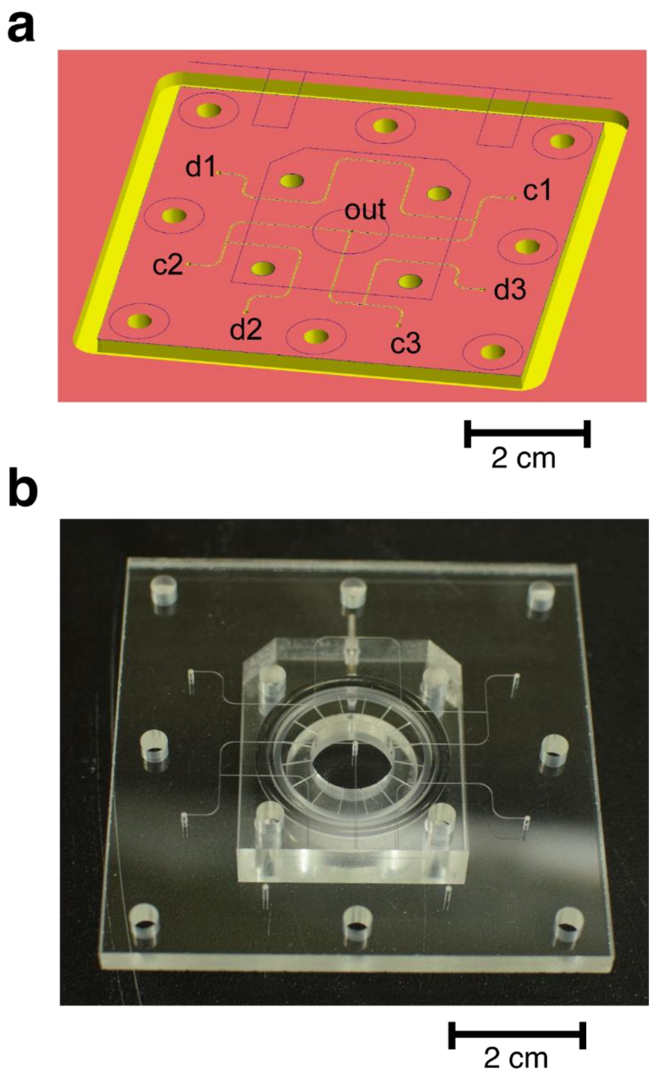

Figure S2. Bottom plate of the cuvette for working with microdroplets. a) Scheme of channels arranged into three microfluidic T-junctions directing droplets to a common outlet – vertical hole in the bottom floor of the cuvette. Channels marked with “d” provide droplet-phase fluid, “c” stands for continuous-phase liquid – typically the same fluorinated oil as the liquid filling the cuvette. **b)** Main plate of the cuvette placed on the plate with T-junctions (in order to seal the assembly, both plates must be separated by a thin sheet of PDMS and tightened together by M4 bolts, not shown here).

Section S2. Image processing and control system

The image recognition, particle tracking and control software were written in Python 3.5 (an outline of the software's structure is provided in **Figure S3**); graphical user interface (GUI) was developed using Kivy library. To improve real-time performance, the software uses multi-threading via Python's native "threading" module and has a total of three threads: main thread which runs the application, a thread used by the image processing module (IMPR) that requests the camera to grab a new frame and retrieves it, and a thread that draws in the GUI an image from the camera with additional graphical elements added by the software. The software operates in a main loop, whose execution is triggered every 33.3 ms, corresponding to 30 frames per second (fps), or when the previous execution of the loop is finished. The software can work either in an automatic regime, when objects are tracked and manipulated without user's intervention, or in a manual regime, when user can manually position the laser beam (as a dot or as a traced shape such as a circle) using GUI. Main controller (MC) within the software is mediating interactions between the GUI, main loop and other elements of the software. MC requests a new frame from IMPR at the beginning of each execution of the main loop; IMPR then reads an image from the buffer of Lumenera Lt425 camera and rescales it from 2048×2048 px to 1024×1024 px (which corresponds to 5.1 mm square field of view), which is being drawn in GUI and, if image-recognition-based manipulation regime is used, used for further processing.

The software supports recognition and tracking of the following objects on the image: disks, squares, triangles, crosses and gear-like objects. For each particle type, if it is used in the ongoing experiment, MC initializes swarm entity that is responsible for organizing particle units. Main functions of swarm include: adding to the list of units until the prescribed maximum limit is reached; arranging the units list by solving Travelling Salesman Problem with Google or-tools

library[1] in order to minimize the eventual laser path; tracking units; deleting from the list when the unit is not detected on the frame for some number of frames (usually set to 10); assigning and keeping the list of units' destinations coordinates. MC requests IMPR to find objects of interest on the most recent camera frame; IMPR then returns center-of-mass coordinates of the detected objects along with vertices of their edge contours. These data are then passed to the swarm that represents detected object type. Upon receiving the data for the first time, swarm initializes one unit object for each detected particle. Each unit has a Kalman filter[2] assigned to it, whose prediction is used by the unit's swarm every subsequent frame to assign particle centers (detected by IMPR in the frame) to unit instances (i.e., to perform particle tracking). To this end, distances between the center of every detected object and coordinates predicted by every Kalman filter are incorporated into a cost matrix, from which the detected-predicted coordinate pairs that minimize the overall cost are selected by solving linear assignment problem with the implementation of the Hungarian algorithm[3] from SciPy library[4].

Thus, the use of Kalman filter-derived coordinates for tracking helps in scenarios with high particle count density and/or movement speed, where simply using the smallest distances between the previous and new positions for mapping objects to unit identities could fail. Moreover, transient lapses in object recognition might cause certain objects to lack assigned coordinates on some frames. As described further down this section, our algorithm is using information of object's state in time to calculate the manipulation parameters – therefore, simply declaring the unit "missing" on the frame when object was failed to be recognized and creating the new unit after it is recognized again would lead to substantially less stable motion control. Using Kalman filter predictions to fill in the coordinates for some frames helps to mitigate this issue. Typically, we consider the unit "missing" after it was not detected for more than 10 frames in a row.

Control Planner module (CP) is used to select a manipulation program, initialize and control the corresponding state machines (SM) and their states for each swarm. SM manages states and switches between them if the state finishing condition is fulfilled (unless CP blocks the state switching, e.g., when another swarm not belonging to the current SM has not yet completed some necessary action). States represent elementary motion tasks, such as “translate to position”, “rotate to specified degree” or “orbit around certain point while rotating around own axis”. If the motion is activated, MC requests CP to perform a movement step. The request propagates from CP to SM, from SM to a state entity, from the state to the corresponding swarm, and from the swarm to its units. Each unit then checks its motion parameters: compares its speed, movement direction, and, if applicable, rotation angle and angular speed with planned values and checks the distance to its destination. After evaluating the manipulation step, each unit returns the contour vertices coordinates (in pixels) and time to move between them to be traced by the laser in order to perform the desired motion. The manipulation contours used are: square for square and circular particles; triangle for triangular particles; trapezoid for cross particles; circle for gears and ellipse for outer planetary gear. Rotation is carried out by rotating the same contours, except for the case of a gear rotation, in which a circle with a “notch” is used (shown in main-text **Figures 1b** and **5d**).

If objects are manipulated by direct heating, the system operates in the following manner. Translational speed of the manipulation contour increases from 5 μm/frame (corresponding to 150 μm/s at full 30 fps performance of the software) up to 40 μm/frame (1200 μm/s at 30 fps) over the distance of 300 μm, then slows down until the full stop starting from 300 μm to the destination point. If unit starts motion for the first time, its manipulation contour size is set to be 1.4 of unit’s physical edge contour size; this value is decreased to 1.1 at a rate of 0.005 per frame. If during unit’s translational acceleration its speed differs from the target value by more than 5 μm/frame –

while the direction of the movement remains towards the destination – the contour's translation speed is changed to the unit's speed and acceleration distance value is set back to the one recorded in a previous movement step. A unit is considered to have escaped the planned motion trajectory if the distance from its physical center to its manipulation contour exceeds 0.5 of the unit's size. In this case, the manipulation contour's translational speed is set to 5 μm/frame, its center is placed to the place it would be after 4 frames if moved with the velocity taken from the Kalman filter's state matrix, and its size is increased to 1.4 of unit's physical size.

The following differences distinguish control procedures if light is absorbed by liquid instead of manipulated objects. Manipulation contour is an arc (approximated by 8-point polygon), whose angle measure α is small when an object is far from the destination, and starts to increase to some final value as the object approaches its destination (see **Figure S4**), according to

$$\beta = \beta_o c_d + \beta_c (1 - c_d) \quad (2.1)$$

$$\alpha = 360° - \beta \quad (2.2)$$

where β is an angle that we term "arc opening angle" (angles are illustrated in **Figure S4**); β_o and β_c are arc opening angles far away and directly at the destination, respectively; c_d is the coefficient given by

$$c_d = \left(\frac{\min\{l_d, l_c\}}{l_c} \right)^{1.7} \quad (2.3)$$

where l_d – distance from unit's center to the destination; l_c – distance to the destination from which arc measure starts to grow. Axis of symmetry of an arc lays on a line connecting arc's center and a destination point; arc's opening faces the destination (see **Figure S4**). Scale of manipulation contour does not change (values can be found in **Table S1**) for hollow glass and white polyethylene

beads and is 1.2 to 1.15 for the brass gear. When the brass gear is rotated, instead of a circle, an arc with a "notch" that heated the vicinity of gear's tooth is used; the "notch" stays at a constant distance from the gear's rotation center, whereas an arc's center is moving together with the gear's center if the gear moves away from the rotation center for more than 55 μm. When many objects are manipulated, distances from each object to the center of mass of the formation is calculated; these distances are normalized between 0.5 and 2 and used as coefficients to the dwelling time of the laser near corresponding objects (in order to reduce the flows in the middle of the formation that push the outer particles away from their destinations). To further reduce these detrimental flows, at the end of each manipulation step the system diverts the laser beam to the point where it escapes the optical path and does not reach the sample plane. The beam stays there for a time equal to the sum of t_{delay} (in μs) values, which are calculated for each object as

$$t_{delay} = \min\{0,7 \cdot d_{obj}\,(1 - c_t\,)\} \cdot 2 \cdot 10^{-5} \tag{2.4}$$

where d_{obj} is the size of the object; c_t is the coefficient given by

$$c_t = (0.7 + c_d)^{2.5} \tag{2.5}$$

See **Table S1** for the values used in the experiments.

In the experiments where four squares are assembled into one larger square (**Figure 4b**), after squares are arranged in the straight line (**Figure 4b**, top-left subpanel) the states that correspond to the assembly steps do not use the manipulation contours returned by units but instead use pre-determined laser traces that heat only the outer (with respect to the center of the unit that is being assembled) edges of the squares.

In the experiments in which microbots control motions of droplets (**Figure 6**, **a** to **d**), microbot destinations are set from the GUI using convenient mouse-controlled drag-and-drop interface; microbots are automatically guided and kept at the destinations.

Swarm entities collect all the manipulation contours-related data from their units, and return it through the call hierarchy from swarms to states, to SM, to CP, and finally to MC, which consolidates, cleans (the points outside the working area of the laser are removed) and sends it to galvo controller (GC) module. It translates the information into a message in a custom-written protocol and sends it via USB to ARM® Cortex®-M7 based STM32F767 microcontroller (STM32F767ZIT6 Nucleo-144 board). It runs a house-written code that outputs voltages via two digital-to-analog converters to control Thorlabs GVS002 galvanometer mirrors; the voltages are updated (thus, the laser is repositioned) every 20 μs.

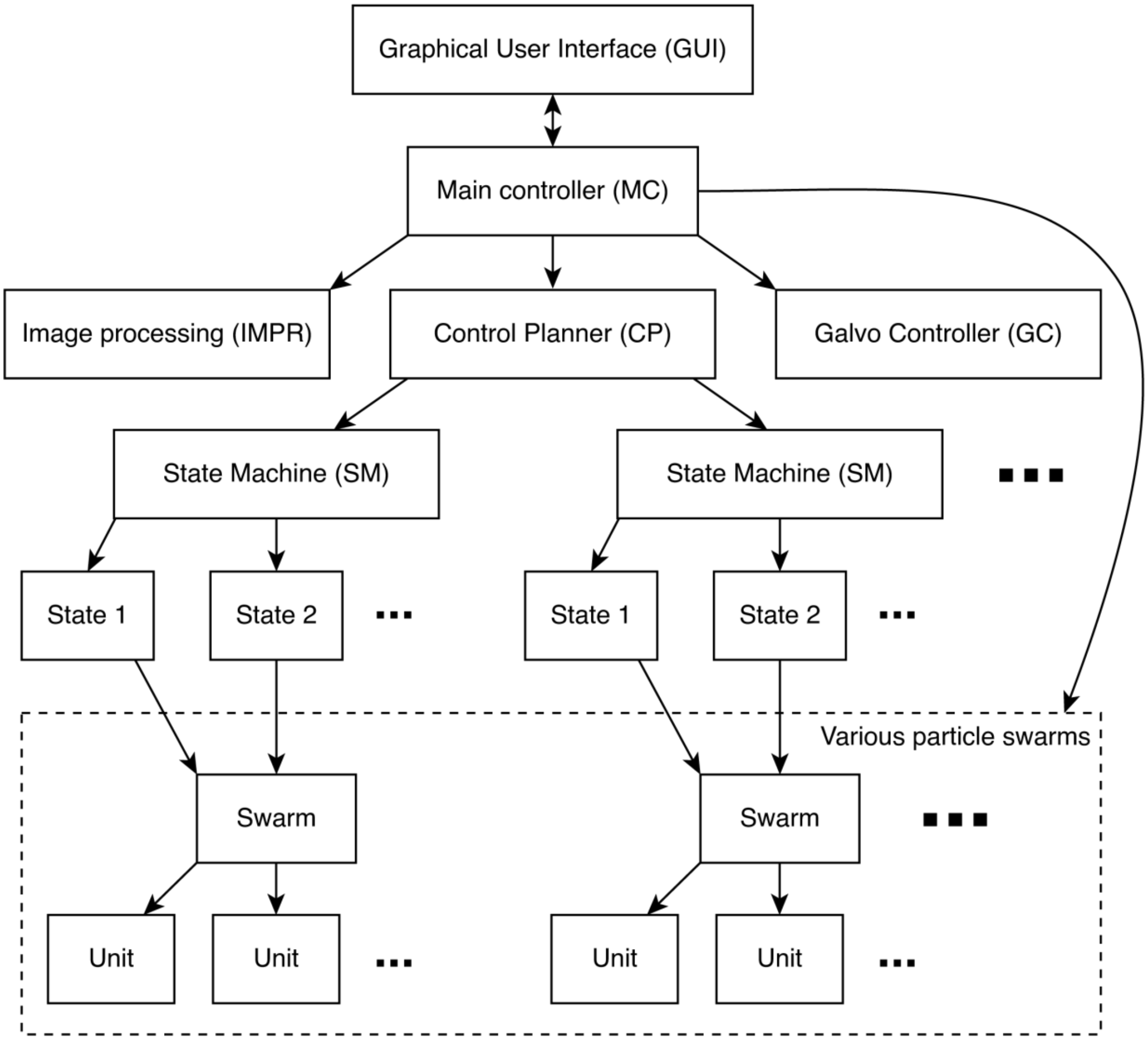

Figure S3. Overview diagram of software's main modules. Entities from which arrows are coming out are calling methods or using attributes of entities to which the arrows are pointing.

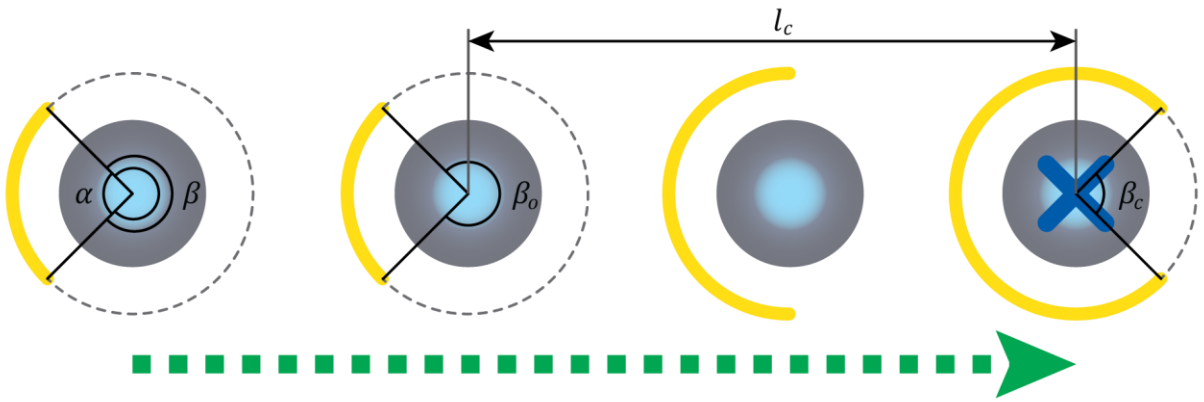

Figure S4. Illustration of arc contour manipulation principle. Yellow arc shows a shape traced by the laser; green dashed arrow indicates direction of motion; blue cross denotes object's destination position; α is the arc measure; β is the arc opening angle, β_o and β_c are arc opening angles far away and directly at the destination, respectively; l_c is the distance from which the arc measure starts to grow as the object approaches its destination.

Table S1. Parameters for manipulation of non-absorptive objects.

	β_o	β_c	l_c (μm)	Arc scale (multiples of object size)
Hollow glass beads, ~120-150 μm	120°	315°	65	3
White polyethylene beads, ~900 μm	60°	300°	150	1.4
Brass gear, 2.7 mm	36°	348.75°	250	1.15 to 1.2

Section S3. Simulations

To explore the physics of light-driven manipulation and obtain insights about the system's dependence on the properties of the phases used, we performed a series of Finite Element Method (FEM) simulations using COMSOL Multiphysics® 5.3a. Below, we first describe simulation setup and then discuss the results (additional to the ones summarized in the main text).

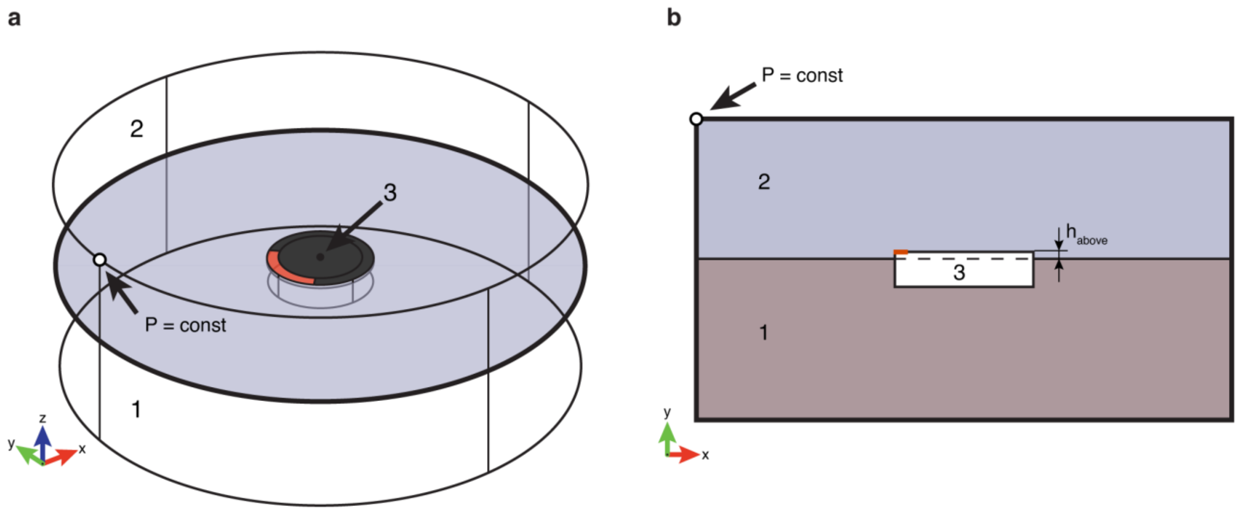

Figure S5. Geometry of simulations. a) Scheme representing simulated region in 3D – a cylinder, divided into 3 subdomains: (1) bottom phase (liquid), (2) top phase (air or liquid) and (3) heated object. Shaded surface represents the interface between domains 2 and 1; red segment on domain 3 shows the heated surface (width 30 μm, matching the laser beam diameter in experiments). **b)** Scheme illustrating the side view in 3D simulations (or the whole geometry in 2D simulations, except the heater's placement); domain representation is the same as in (**a**). Parameter h_{above} is the height of the object's part that resides in the upper phase (2).

Simulations' configuration

3D simulations were performed with a static heated object. The "global" simulation domain (**Figure S5**) was a cylindrical chamber 15 mm in diameter and 6 mm in height, similar to the real chamber used in experiments. This cylinder was divided into three subdomains. The lower region (1) was treated as filled with FC-40 liquid, as used for manipulations in most experiments, whereas the upper domain (2) was either air or Shin-Etsu KF-96L-5cs Silicone oil (whose interfacial surface tension with respect to FC-40 has been measured experimentally as a function of temperature[5]); horizontal interface between them was placed in the middle of the global simulation domain. The manipulated PDMS object (0.3 mm in diameter and 0.08 mm in height) was represented by a solid body placed at the center of the interface between these two regions (domain "3" in **Figure S5**).

To set the position of the particle with respect to the interface in the simulations, we used the following reasoning. In experiments we observed that the interface was at the submerged object's bottom edge in liquid/liquid scenario or at the top edge in liquid/gas configuration. This positioning is due to PDMS being philic to FC-40 (in the bottom phase), but more philic to silicone oil or decane (in the upper phase)[6]. The angle θ between the interface and sides of the submerged object was estimated considering buoyancy, gravitational and capillary forces (F_b , F_g and F_c , respectively) acting on the cylindrical particle in equilibrium:

$$F_g + F_b + F_c = -\pi\tau R^2 g\rho_{object} - \pi\tau R^2 g\rho_{liquid} + 2\pi R\sigma \cdot \cos(\theta) = 0 \qquad (3.1)$$

which reduces to

$$\cos(\theta) = \frac{(\rho_{liquid} - \rho_{object})\tau R g}{2\sigma} \qquad (3.2)$$

where: θ – contact angle; R and τ – radius and height of the cylinder, respectively; g – gravitational acceleration constant; σ – surface tension coefficient; ρ_{liquid} and ρ_{object} – densities of the liquid and the object, respectively. The vertical position of the particle was determined by the height of the meniscus at the particle's side, which was evaluated by solving the Laplace equation[7]:

$$-\rho_{liquid} g z = \sigma(\frac{1}{R_1} + \frac{1}{R_2}) \quad (3.3)$$

where: z – the height of the liquid surface (with respect to the height at infinity); r – radial distance from the center of the object; R_1, R_2– main surface curvatures. For the geometry used, the explicit form of the equation (3.3) is:

$$\frac{\rho_{liquid} g z}{\sigma} = \frac{z'}{r} + \frac{z''}{(1+(z')^2)^{\frac{3}{2}}} \approx \frac{z''}{(1+(z')^2)^{\frac{3}{2}}} \quad (3.4)$$

where: z', z'' - first and second derivatives with respect to r; [8]; the shape of the surface is axisymmetric since the particle is cylindrical. We solved equation (3.4) numerically in MATLAB ver. 2019a using fourth-order boundary value problem solver (assuming zero surface elevation at the long distance from the particle). The calculated value of the contact angle θ was 89.8^o, while the height of the meniscus induced by the object to the interface at rest was found to be 0.96 μm, which is on the order of 1% of the total height of the object. In order to treat simulation stability issues and to keep the mesh size reasonable, we fixed the value of the contact angle at 90° and used 5% to 95% submergence proportions – when liquid/air configuration was simulated h_{above} (**Figure S5**) was 5% (4 μm), while in simulations of liquid/liquid configuration h_{above} was 95% (76 μm). The phase-separating interface was set to be flat initially (because of the relatively small capillary

height); due to the simulations configuration the interface shape changed as the simulations proceeded, albeit negligibly.

We used two COMSOL Multiphysics® modules in all studies: "Heat Transfer" (HT) and "Laminar Two-Phase Flow Moving Mesh" (TPFMM). TPFMM was solving Navier-Stokes equation (3.5) with Continuity equation (3.6) in domains 1 and 2, assuming all of the simulated liquids and air were incompressible:

$$\rho\frac{\partial \boldsymbol{u}}{\partial t}+\rho(\boldsymbol{u}\cdot\nabla)\boldsymbol{u}=\nabla\cdot[-\rho\boldsymbol{I}+\mu(\nabla\boldsymbol{u}+(\nabla\boldsymbol{u})^{T})]+\boldsymbol{F} \tag{3.5}$$

$$\rho\nabla\cdot\boldsymbol{u}=0 \tag{3.6}$$

where: $\boldsymbol{u}$ – flow velocity field; ρ – density; $\boldsymbol{I}$ – identity tensor; μ – viscosity, and $\boldsymbol{F}$ denotes gravitational force on liquids. Calculations were performed using first-order discretization. The reference pressure was set to be 1 atm; we applied the "Pressure Point Constant" node on the point shown in **Figure S5** for pressure field determination. TPFMM module used the following boundary conditions: no-slip boundary condition on the top and bottom edges of domains 2 and 1, as well on all sides of domain 3. For the appropriate calculation of the moving mesh, Navier-slip boundary conditions (3.7) and (3.8) were applied on the sidewalls of the global domain.

$$\boldsymbol{u}\cdot\boldsymbol{n}=0 \tag{3.7}$$

$$\boldsymbol{n}\cdot\boldsymbol{T}=-\frac{\mu}{\beta}\boldsymbol{u} \tag{3.8}$$

where: $\boldsymbol{n}$ – normal vector to the surface; $\boldsymbol{T}$ – liquid stress tensor; β – slip length parameter (chosen by default in the software). Following equations were applied on the boundary that separates domains 1 and 2:

$$\boldsymbol{u}_1=\boldsymbol{u}_2 \tag{3.9}$$

$$\boldsymbol{n}_1 \cdot \boldsymbol{T}_1 = \boldsymbol{n}_2 \cdot \boldsymbol{T}_2 = \sigma(\nabla_t \cdot \boldsymbol{n}_1)\boldsymbol{n}_1 - \nabla_t \sigma \quad (3.10)$$

$$\boldsymbol{u}_{\boldsymbol{mesh}} = (\boldsymbol{u}_1 \cdot \boldsymbol{n}_1)\boldsymbol{n}_1 \quad (3.11)$$

where lower indices denote the domain of origin for each value; ∇_t – derivative along tangential direction; σ – surface tension between domains 1 and 2.

Dependence of σ on the temperature was governed by equation (3.12):

$$\sigma = s_0 + c(T - T_0) \quad (3.12)$$

where s_0, c and T_0 are parameters that depend on a choice of phases (for FC-40/KF-96L-5cs interface $s_0 =$ 5.811 mN/m and c = −0.0382 mN/m/K; for other values see **Table S2**); T – temperature obtained from coupled solutions of the heat transfer equations using the HT module.

The mesh movement was computed using the "Prescribed mesh displacement" node in the TPFMM module along the z-direction on the lateral edges of the global boundary as well as the lateral boundary of domain 3. These settings were necessary to use the TPFMM module, which allowed us to compute interactions between two liquid phases. Over the remaining boundaries, mesh displacement was fixed to zero along all coordinate axes.

HT module was solving heat transfer equations (3.13) and (3.14) in all domains:

$$\rho C_p \frac{\partial T}{\partial t} + \rho C_p \boldsymbol{u} \cdot \nabla T + \nabla \boldsymbol{q} = Q \quad (3.13)$$

$$\boldsymbol{q} = -k \nabla T \quad (3.14)$$

where: $\boldsymbol{u}$ – velocity field from the TPFMM module; Q – external heat, represented in the system by applying a "Boundary Heat Source" node on the surface which is marked red in **Figure S5**. For equations (3.13) and (3.14), Dirichlet boundary conditions were used, so that temperature $T_{external}$ was constant on all boundaries of the global domain. The value of $T_{external}$ as well as initial temperature in the whole system were set to 293.15 K.

Analyzing the results of each simulation, we calculated the force on the object imparted by thermocapillary-induced flows and surface tension gradients. This force was estimated by integration of the fluid stress tensor $\boldsymbol{T_{ij}}$ along the boundary of domain 3 and summed with edge integral of the quantity $\sigma \boldsymbol{n}$ along the contact points between the body and the interface.

2D Simulations were performed to study properties of object's motion in various liquid/air configurations. Simulations were performed in 2D to make feasible the modelling of a wide variety of configurations (225 in total; 25 sets of initial conditions for 9 different liquids) within a reasonable computation time. We added "Moving Mesh" (MM) and "Global ODEs and DAEs" (GE) modules to the model; other modules and their options were the same as in 3D case, except the changes described below. The region of the simulation in 2D was divided into subdomains (1 and 2 in **Figure S5b**) in a way equivalent to the 3D simulations; the boundary conditions were also similar. Instead of one heated region, two heaters were placed as shown in **Figure S6a**. These heaters acted upon the particle's top surface (domain 3) when the particle was below a given heater. The amount of heat applied in each simulation step was calculated from the heater's power density and the intersection length.

GE node was solving the equation of motion of the moving particle/domain 3:

$$m\frac{dv}{dt} = F;\ \frac{dx}{dt} = v \tag{3.15}$$

where: x –the displacement magnitude of the domain 3 along the x-axis (**Figure S5b**); v – velocity of this region along the same direction; F – total force acting on the particle) along x-axis. MM module was used to introduce dynamical mesh displacement of the particle. The value of x was used in the "Prescribed Deformation" node as rate of displacement along x axis.

For the simulation to work properly, we changed the no-slip boundary condition in the “Wall” node of the TPFMM module, applied to all boundaries of the domain 3. Namely, unlike in 3D case, we used a relation $u = -v$, where v is the speed of domain 3, obtained from (3.15) and u is the projection of the fluid velocity field $\boldsymbol{u}$ on the x axis; it allowed us to obtain correct calculations of the fluid velocity field. Prescribed displacement along the x-axis in the “Prescribed Mesh displacement” node, applied on the boundary of region 3, was set to the value of x (3.15).

Geometrical dimensions were set as follows: global simulated region was rectangle of 16 mm width and 9 mm height, divided into two domains in the middle; domain that represents manipulated particle was also a rectangle with height and width equal to height and diameter of the corresponding domain in 3D simulations – 0.08 mm and 0.3 mm, respectively. The “Thickness” setting in HT module, which represents “depth” – dimension, perpendicular to the simulated plane – was set to 0.3 mm.

Table S2 | Parameters of liquids used in simulations.

	Density (kg/m^3)	Thermal conductivity (W/m/K)	Viscosity (Pa·s)	Heat capacity (J/kg/K)	c (mN/m/K)	s_0 (mN/m)
FC-40	1870	0.067	0.0041	1050	−0.0862	18.787
FC-70	1940	0.071	0.024	1050	−0.0799	20.319
Water	998	0.598	0.00089	4181.6	−0.1396	75.668
Hexane	613	0.12	0.0003	2293	−0.10267	20.408
Decane	730	0.147	0.00085	2217	−0.09429	26.341
Novec 7200	1430	0.068	0.00061	1214.1	−0.08913	15.866
KF-96L-5cs	915	0.12	0.00458	1758	−0.0734	21.4
KF-96L-20cs	950	0.15	0.019	1632	−0.0796	22.84
Novec 7500	1614	0.065	0.00128	1128	−0.07206	16.938
Hexadecane	770	0.146	0.00305	2206.7	−0.0809	29.11

Discussion of simulation results

A liquid ideal for particle manipulation should provide some optimal combination of movement speed and control. We probed these qualities by simulating a thermocapillary "trap" in 2D, in which the particle moved between two flanking positions of the laser beam heating the particle's edges and thus setting up thermocapillary flows (**Figure S6a**). We assumed that particle escaped if its center's displacement from the middle of the "trap" > 200 μm and stabilized if the displacement was less than 1 μm with the speed slower than 150 μm/s; simulation was stopped if the particle settled, escaped, or did not settle or escape within 1 second. We simulated motion in: FC-40, FC-70, Novec 7200 and Novec 7500 fluorinated solvents; Shin-Etsu KF-96L-5cs and KF-96-20cs silicone oils; hexadecane, decane and water. A set of 25 initial parameters was used for each liquid: combinations of 5 starting displacements x_0 and 5 heating levels (**Figure S7**). Heating power Q was used to derive a heating power density as power Q over one squared unit of "Thickness" of HT module (300×300 μm^2). In this way, Q values of 0.1, 0.5, 1, 5 and 10 mW correspond to power densities of 0.(1), 0.(5), 1.(1), 5.(5), and 11.(1)W/cm^2, respectively, and to each heater providing 10, 50, 100, 500 and 1000 μW of power, respectively.

The 2D situation in which the trapped particle – being heated "from the left" to move "right" and then "from the right" to move "left" – is analogous to an oscillator having a linear-regime region around the center of the trap (**Figures S6** and **S7**). Given the harmonic oscillator equation:

$$m\ddot{x} + b\dot{x} + kx = 0 \qquad (4.12)$$

we extracted parameters $\beta = \frac{b}{m}$ and $\kappa = \frac{k}{m}$ by fitting a plane to the linear region in the simulated coordinate-velocity-acceleration phase space (**Figure S6b**, points of the phase trajectory were classified as belonging to the linear region if they were within 20 μm around the center of the

“trap”; if that range did not encompass at least 6 points of the curve, it was expanded). Parameters κ and β are analogous to elastic (spring) and damping coefficients, respectively: coefficient κ is growing with increasing heater power and, thereby, with forces that are pushing the particle, but does not depend on the initial displacement (**Figure S6c**); coefficient β is negative when the particle escapes from the “trap” (the system is unstable), close to zero when the particle keeps oscillating (the system is marginally stable), and positive when the particle stabilizes inside the trap (the system is damped) (**Figure S6d**).

Based on the inspection of β and κ averaged over 25 simulations for each liquid, FC-40 appears to be optimal for thermocapillary manipulations of sum-mm and larger objects. In the absence of a well-defined cost function to find optimal combination of parameters, we used principal component analysis (PCA) to quantitatively asses this assumption (**Figure S8**). PCA removes the correlation between β and κ by translating points to a new basis (**Figure S8a**, the data after PCA is on the right). We denote “Manipulation score” the value of the second PCA basis (inverted to match the direction of optimality of the parameters) since the highest point along that basis represents the point with the best tradeoff between β and κ. Consistent with our observations, FC-40 has the highest score.

Using the data from the simulations, we assessed the contribution of the most relevant properties of the liquids: heat capacity, thermal conductivity and viscosity. The motion originates from the thermocapillary stress at the surface, given by:

$$\tau = \mu \frac{d\vec{u}}{dn} = -\gamma \nabla T \quad (4.13)$$

where $\frac{d\vec{u}}{dn}$ is the gradient of horizontal fluid velocity normal to the surface, $\gamma = \frac{d\sigma}{dT}$ is the coefficient of surface tension dependence on a temperature, and ∇T is the surface temperature gradient;

therefore low thermal conductivity is beneficial for the creation of larger ∇T upon heating, as demonstrated by the manipulation score in **Figure S8b**. High heat capacity tends to be, in general, detrimental to efficient manipulation (**Figure S8c**). It can be attributed to the fact that the bigger the heat capacity, the more energy has to be pumped in the system by heaters, which takes longer time. Contribution of viscosity is less straightforward: it influences motion characteristics through both the induced thermocapillary stress (as can be seen from equation (4.13), increase in viscosity leads to increased stress) and dampening properties of the liquid. Particle stability, represented by β, worsens with decreasing liquid viscosity (**Figure S8f**); concurrently, parameter κ – which characterizes the promptness of response to the heating – decreases when liquid viscosity increases (**Figure S8g**). These considerations imply existence of some optimal region of viscosity, which can be seen in **Figure S8d**: two liquids, FC-40 and KF-96L-5cs silicone oil, having similar viscosity (4.58 cS and 4.1 cS, respectively), also have the highest manipulation scores, which matches our experimental observations for the sub-mm and larger particles (for very small colloids, more viscous FC-70 ensures more control over particles' motions). Dimensionless Prandtl number combines the three described parameters:

$$Pr = \frac{c_p \mu}{k} \tag{4.14}$$

where c_p is heat capacity and k is thermal conductivity, and also demonstrates a pattern with an optimal region (**Figure S8e**).

We further investigated how different parameters of a liquid influence the particle's motion parameters using 3D simulations of static object at a liquid/air interface (as described before), but this time instead of changing liquids, we were changing one parameter of the liquid (viscosity, heat capacity or thermal conductivity), while keeping the rest equal to the native FC-40 values

(**Figure S9**); the heating power was 1 mW. These simulations show that, as expected, the force that acts on the object decreases with increasing heat capacity and thermal conductivity (**Figure S9**, **a** and **b**), and increases when viscosity grows (**Figure S9c**). Moreover, these simulations revealed one of the reasons for the existence of optimal viscosity region: the increase of the force with viscosity is approximately logarithmic; at the same time, on the scale of interest, the growth of viscous drag is linear. Hence, after some viscosity value, the benefit of increased force is outmatched by the detrimental effects of the drag. As can be seen in **Figure S9d**, the rate of increase in force drops rapidly after viscosity passes the native value of FC-40.

Simulations of the FC-40/air system in 3D with 0.3 mm disk (also shown in the main text **Figure 2**, **a** and **c**) revealed that the force on the particle, maximal flow speed in the liquid (**Figure 6c**), and maximal temperature on the object (**Figure S10b**) grow approximately linearly with heating power. Temperatures close to maximal occur only in the region where heat is applied, dropping sharply in its vicinity, as can be seen in **Figure S10a** (see also experimental temperature profiles in **Figures S10c,d**). When diameter of the disk was changed, while thickness, heating power, heating zone width and its arc measure remained the same (emulating the same control approach with the same laser parameters in "real" system), the simulations showed that force growth is linear at first but slows down when the disk diameter becomes larger than ~500 μm (**Figure S11**).

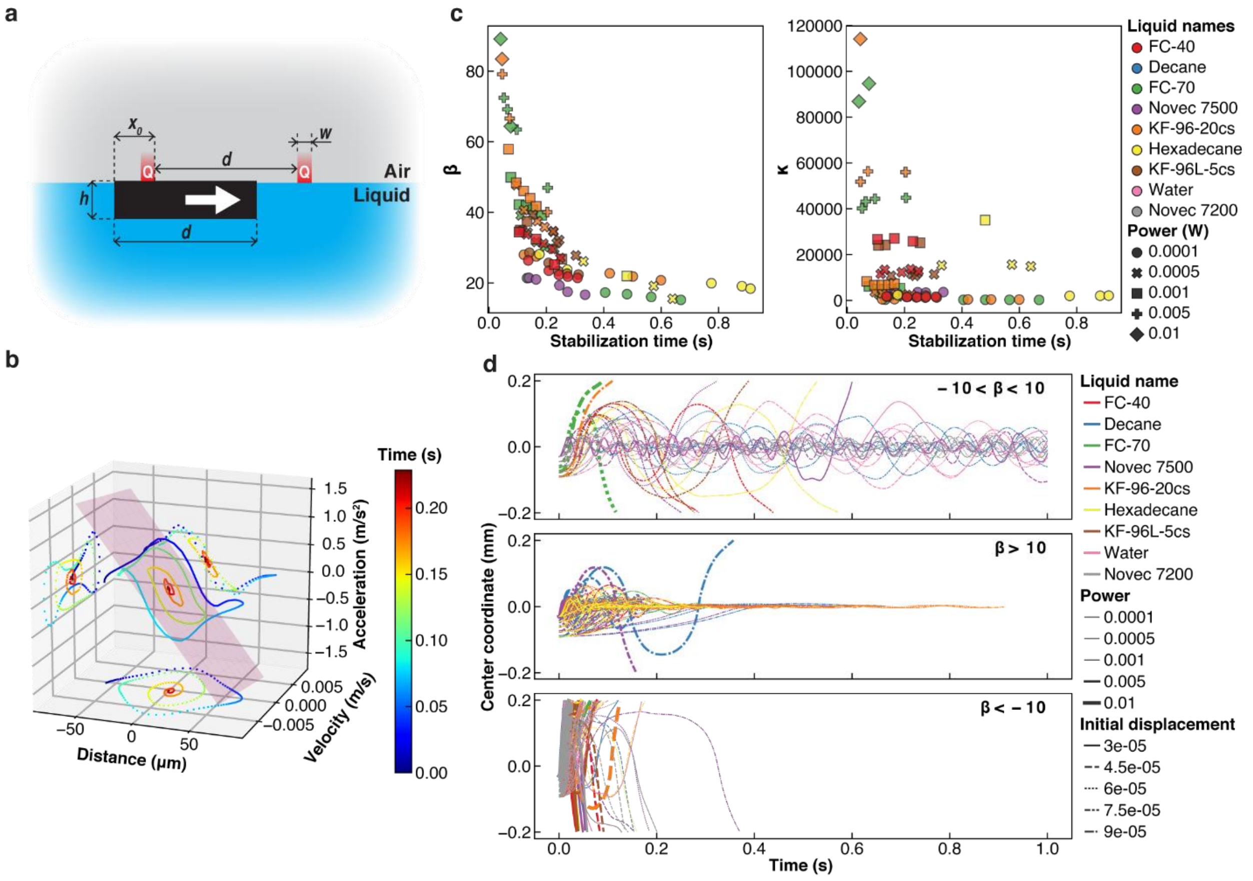

Figure S6. 2D simulations of a thermocapillary "trap". a) Scheme of a d = 300 μm and h = 80 μm PDMS particle initially displaced by x_0 (varied) from the center of the trap. The particle's top surface is heated over the regions corresponding to 30-μm-wide "heaters"/laser beams. White arrow denotes particle's direction of motion – here, because the left laser beam is impinging on the left portion of the particle, the thermocapillary flows are such that the movement is to the right. The particle is nearly completely submerged such that only upper 4 μm are in the air phase. **b)** Example of fitting a plane (in magenta) to the linear oscillator region of the phase trajectory from the simulation in FC-40 with x_0 = 60 μm and Q = 1 mW. Dotted lines are projections of the phase trajectory on 2D planes. **c**) Dependence of β (left plot) and κ (right plot) on the stabilization time. Values are shown only for the simulations in which the particle ends up stabilized inside the trap. Markers of the same shape and color represent different x_o. **d**) Coordinate of the particle's center

in time for -10 < β < 10 (top), β > 10 (middle) and β < -10 (bottom). All simulated trajectories are shown.

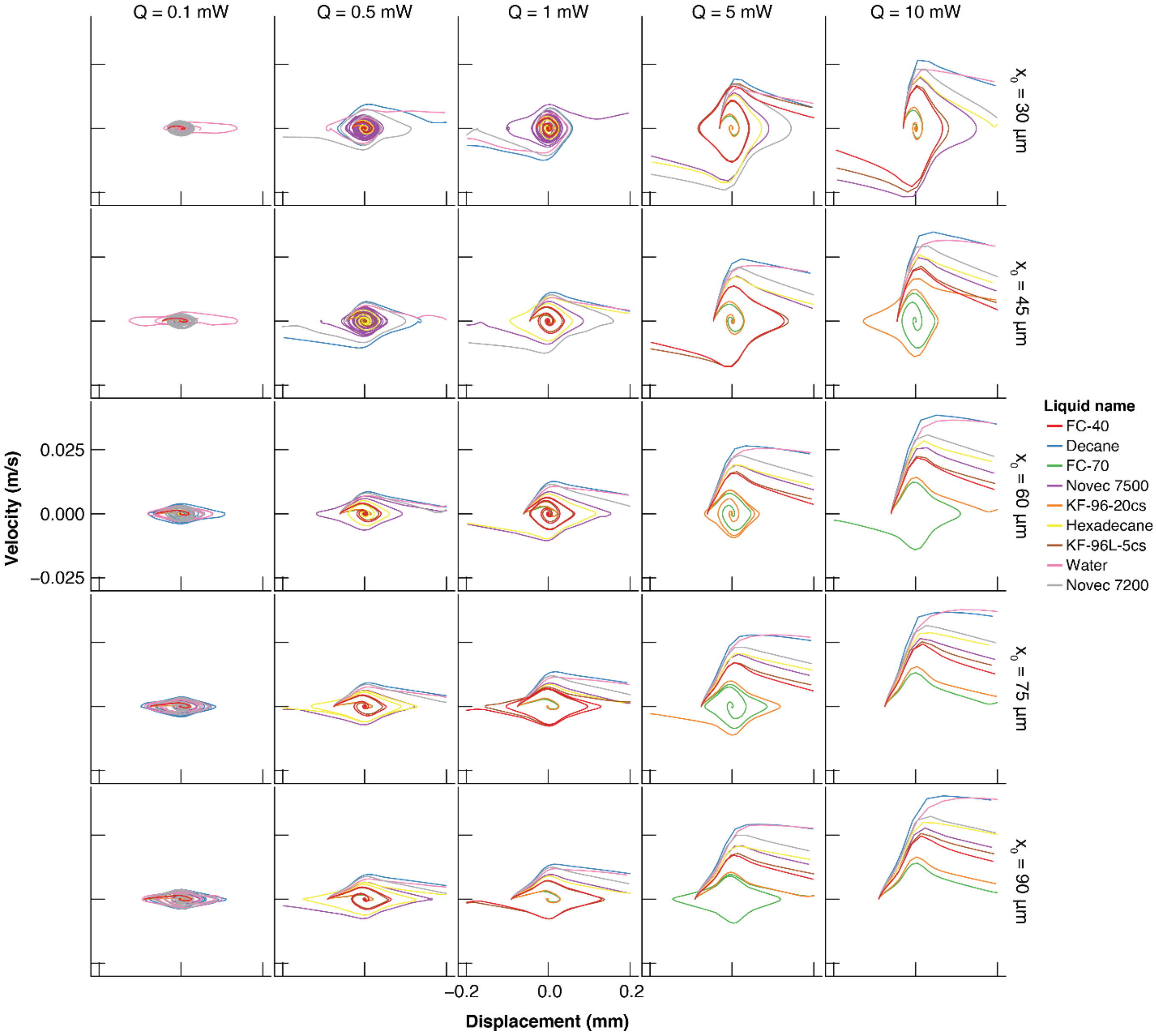

Figure S7. Phase trajectories of the particle for 2D "trap" simulations. Across all plots, horizontal and vertical axes share the same dimensions, respectively. Columns correspond to the same heating power *Q* (labeled on the top of a column), rows correspond to the same initial displacement x_0 (labeled to the right of each row).

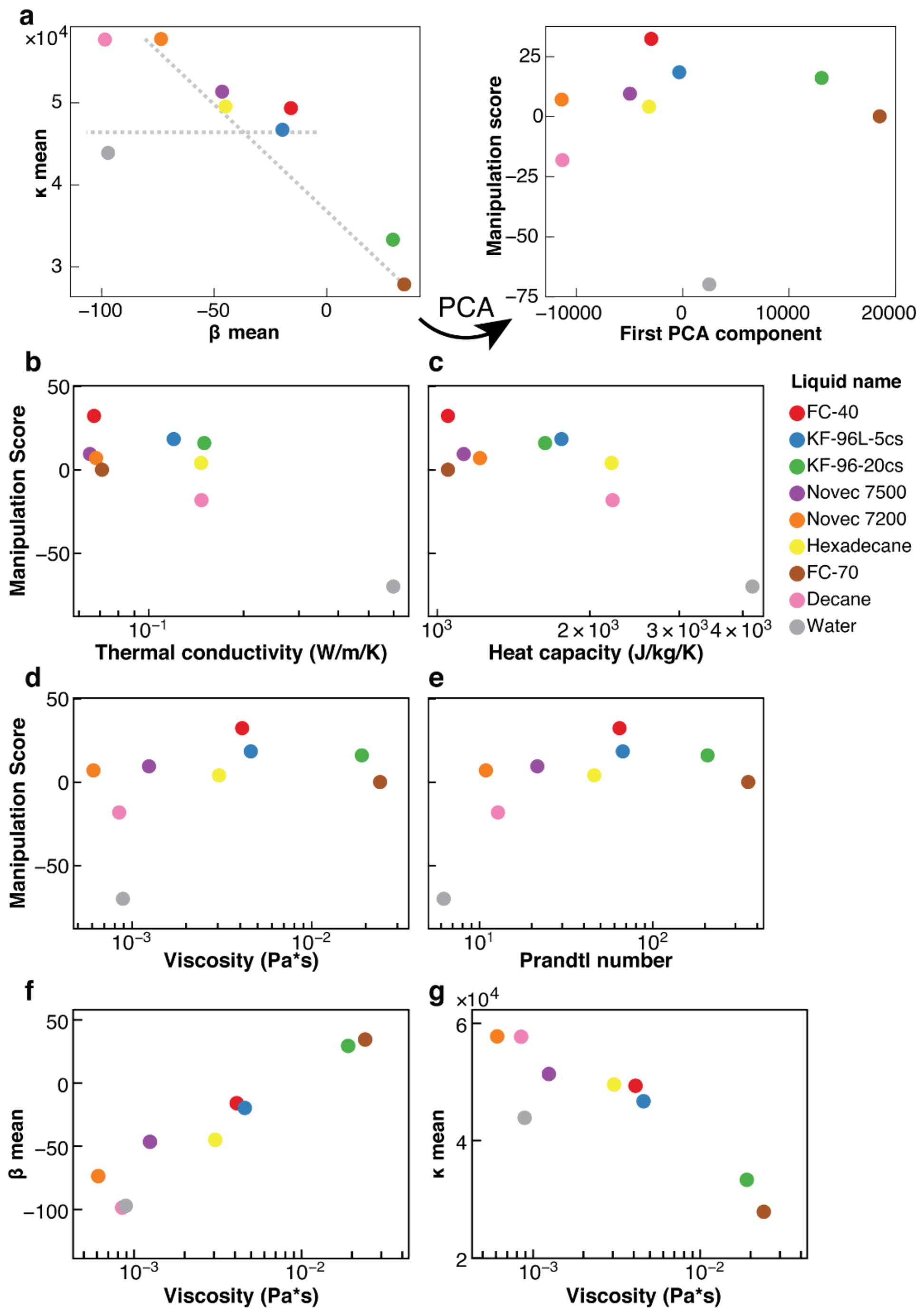

Figure S8. Analysis of "manipulation score" from 2D trap simulations. a) Data before (*left*) and after (*right*) PCA transformation. **b-e)** Dependence of the manipulation score (defined in the main SM text) on various parameters of the liquids. **f,g)** Dependence of β and κ on liquids' viscosities.

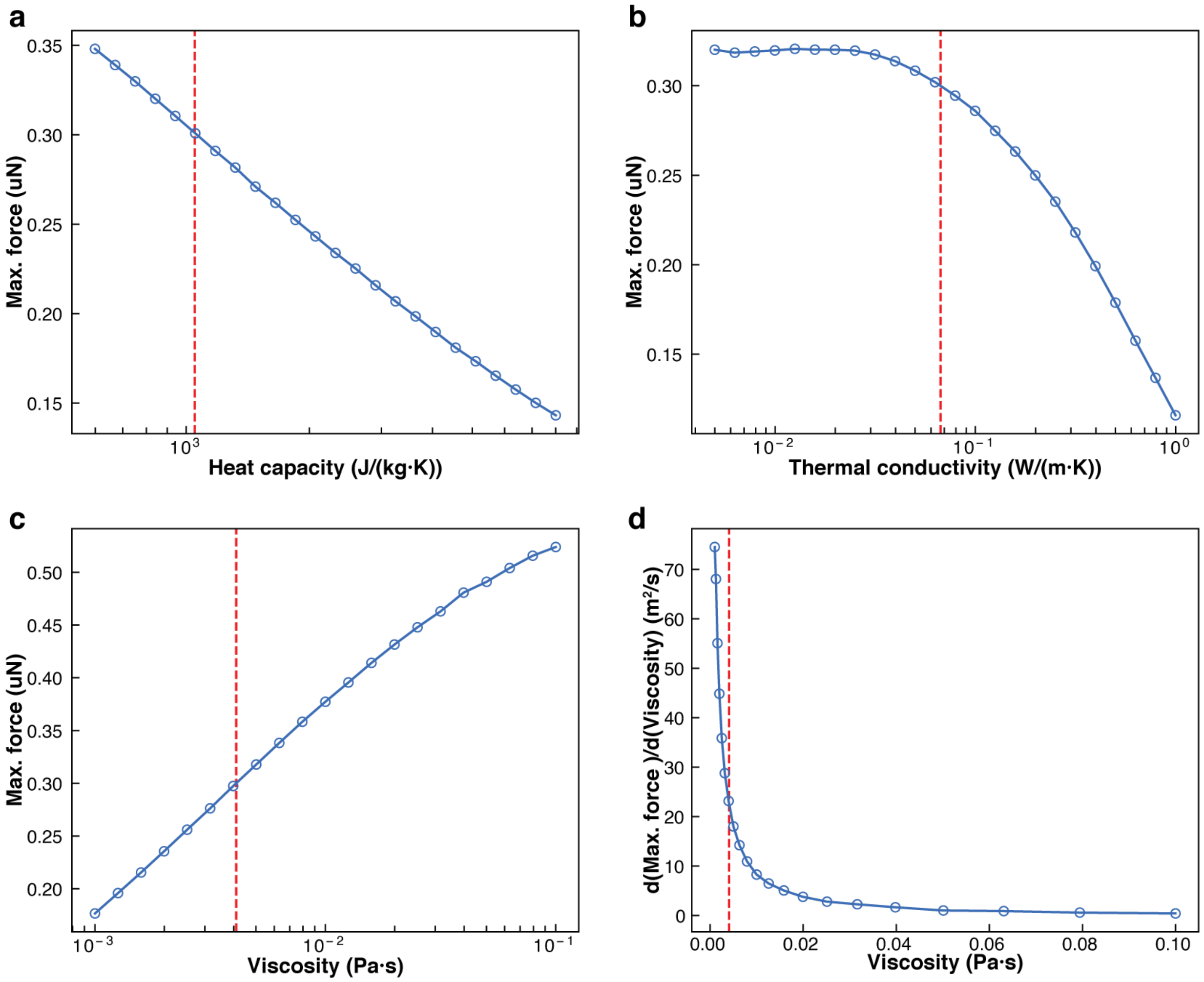

Figure S9. Analysis of maximal force when one of parameters of FC-40 is varied. In **a-c**) heat capacity, thermal conductivity or viscosity is varied, respectively. **d**) Rate of change of maximal force with viscosity. Red dashed line indicates native values of FC-40 parameters.

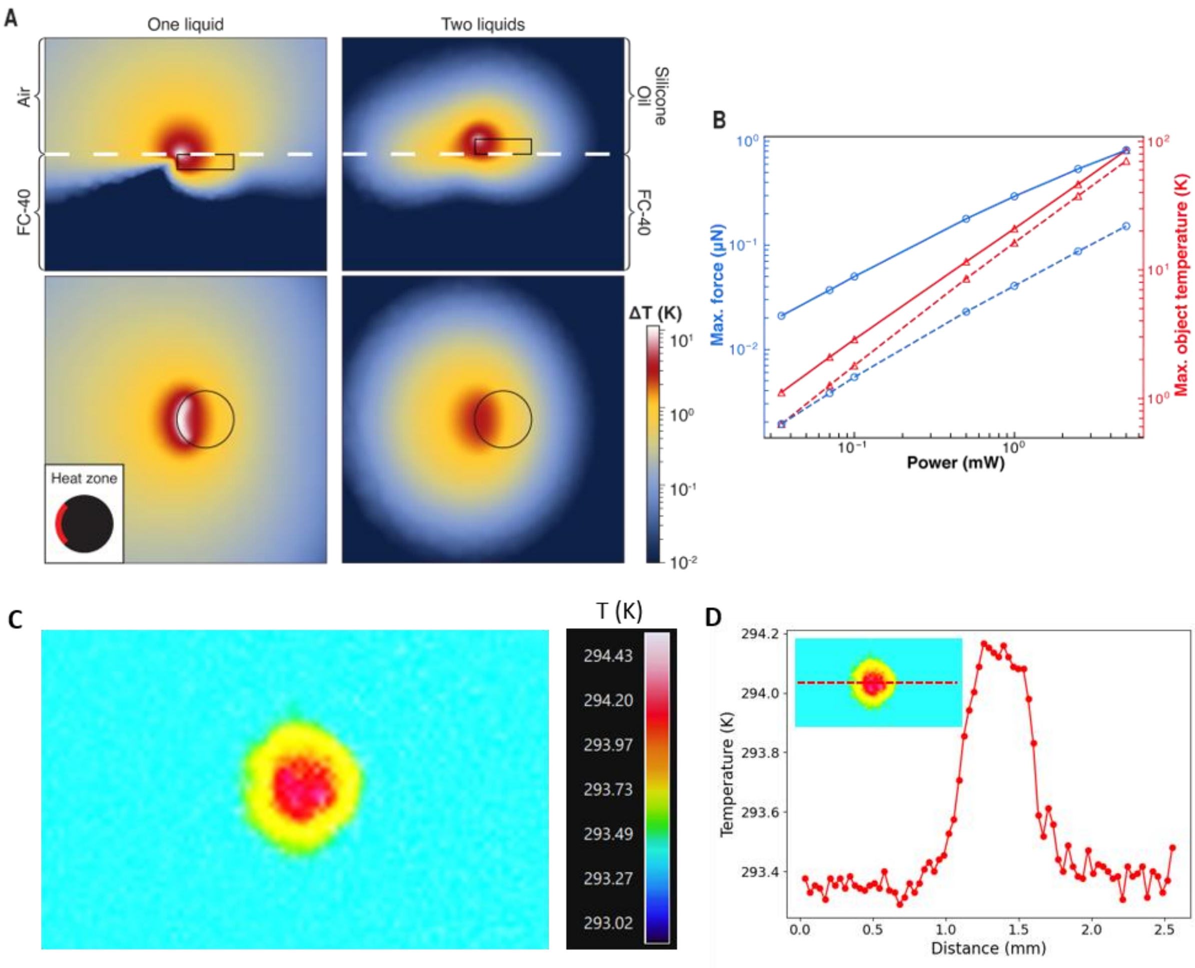

Figure S10. (a, b) Results from 3D simulations and (c, d) experimental temperature profile. a) Temperature map from simulations for a 0.3 mm disk and FC-40 as the bottom liquid (data from the simulations is also shown in the main-text **Figure 2a**,**b)** Plots of maximal force and of maximal object temperature when heating power is varied, data from these simulations are also shown in the main-text **Figure 2c**. **c)** Experimental temperature distribution over a FC-40/air interface housing polyethylene microbeads (BKP-MS-1.2 850-1000 μm, Cospheric) coated with a black dye. The particles were irradiated with 635 nm, 1 mW laser and the thermal image was taken with an IR camera (FLIR A8300sc). **d)** Temperature profile along the dashed red line shown in the inset. Temperature difference between the particle and the surrounding liquid is ~1 deg.

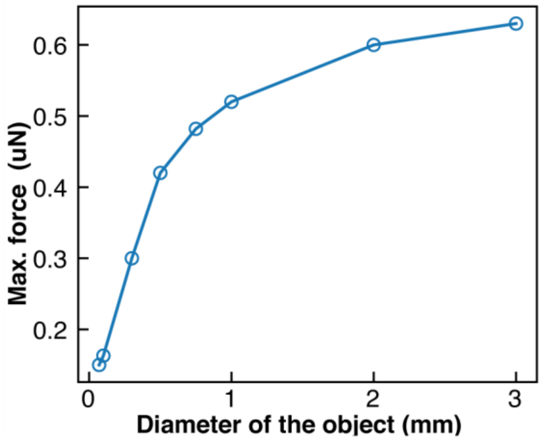

~

Figure S11. Scaling of maximal force with disk diameter. Heating power was 1 mW for all datapoints.

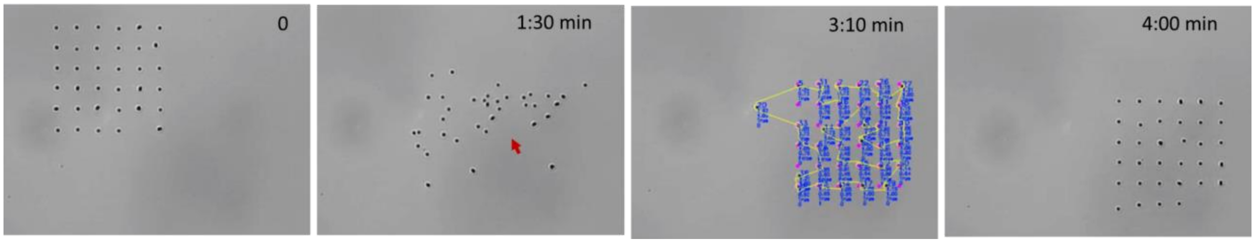

Figure S12. Re-formation of a 6×6 square lattice of 1.43 μm polystyrene microbeads at a new location of a hexadecane/FC-40 interface (see main-text **Figure 3** and **Video S1** for the assembly of such particles into larger, 14×14 lattices). The left-most image shows the lattice already assembled at some initial position. This lattice is allowed to disassemble and the red arrow in the second panel defines a new position around which the particles are supposed to re-assemble. The third panel illustrates the working of the algorithm: yellow lines represent laser traces, blue curves represent particle tracking, and pink dots correspond to the pre-programmed target about which the particles will assemble to re-form the square lattice (shown in the rightmost image).

Section S4. Experimental measurement of force

In order to validate the force magnitude obtained from the simulations, we measured the forces generated in our system by experiment. We placed a black PDMS disk (diameter 300 μm, height 80 μm, as in 3D simulations) on the convex surface of the FC-40 (obtained by pinning the liquid along the circumference of the polycarbonate chamber 12 mm in diameter) as shown in **Figure S13a**. The chamber was sealed to prevent evaporation of FC-40 to the atmosphere (thus preserving the interface position and shape); sealing cap had a window on the top for microscopic imaging and laser delivery and a window on the side to allow imaging with Nikon D850 DSLR camera (**Figure S13a** has the view through the side window). Upper window of the outer chamber was weakly heated from the outside with a nichrome wire loop in order to prevent fogging up of the window and to avoid variation of the laser power that passes through it. The PDMS disk was made

to move by the laser beam (shape of the laser trace shown in yellow color in **Figure S13c**) to the position on the curved surface of the liquid close to the wall of the chamber (see scheme in **Figure S13b**). With laser power fixed, we pushed the particle closer to the wall until any further attempt to reduce the distance between the wall and the particle L (**Figure S13, b** and **c**) would result in either movement of the laser only (the particle stays in place and cannot be "pushed" forward) or escape of the particle from the laser. The distance L depended on the power of the laser – the more powerful it was, the smaller was the distance. The position reached by the particle was determined by the balance of forces caused by buoyancy, gravity, and thermocapillary effects. The force balance for projections of the forces on the interface is given by:

$$F_T = F_b \sin(\theta) - F_g \sin(\theta) \tag{5.1}$$

where F_b is the buoyancy force, F_g is the gravitational force, F_T is thermocapillary induced force, and θ is an angle of inclination of the tangent to the liquid surface in object's center with respect to horizontal plane (axes τ and x on **Figure S13b**, respectively). Since the distance L is minimized for a given laser power, we assume that the resulting thermocapillary force F_T is maximal under this power. The values for θ were obtained from graphical analysis of the side photos (e.g. **Figure S13a**) at each particle's position, which was determined from the top view (e.g., **Figure S13c**). The force F_{bg}, which results from buoyancy and gravity, was calculated as:

$$F_{bg} = F_b - F_g = V \cdot (\rho_{liq} - \rho_{obj}) \cdot g \tag{5.2}$$

where V is the volume of the particle (which is practically completely submerged, see **Section S3** for the details on particle's placement on the interface); ρ_{liq} and ρ_{obj} are densities of liquid and PDMS, respectively; g is the standard gravity; the calculated value of F_{bg} was ~49 nN. Since upon

decreasing L the angle θ grows, projection of F_{bg} on the interface also grows, which allows us to calculate different thermocapillary forces as $F_T = F_{bg}\sin(\theta)$.

In the described experimental configuration, it is practically inevitable that some laser light will pass by the particle without hitting it – accordingly, we measured that transmitted power under the experimental chamber (which is transparent) and subtracted it from the laser power at the sample plane to obtain an estimate of power absorbed by the particle (assuming all the light that falls on the particle was absorbed).

It is important to note that the method described is not without limitations. First, as can be seen from equation (5.1), it is impossible to measure the value of F_T greater than the buoyancy force F_b. Second, the presence of the chamber wall in the particle's vicinity and curvature of the liquid surface both influence the flows in the liquid and, therefore, the obtained force magnitude. In view of this, we used only relatively small laser powers for the force measurements (35, 70 and 100 μW on the particle).

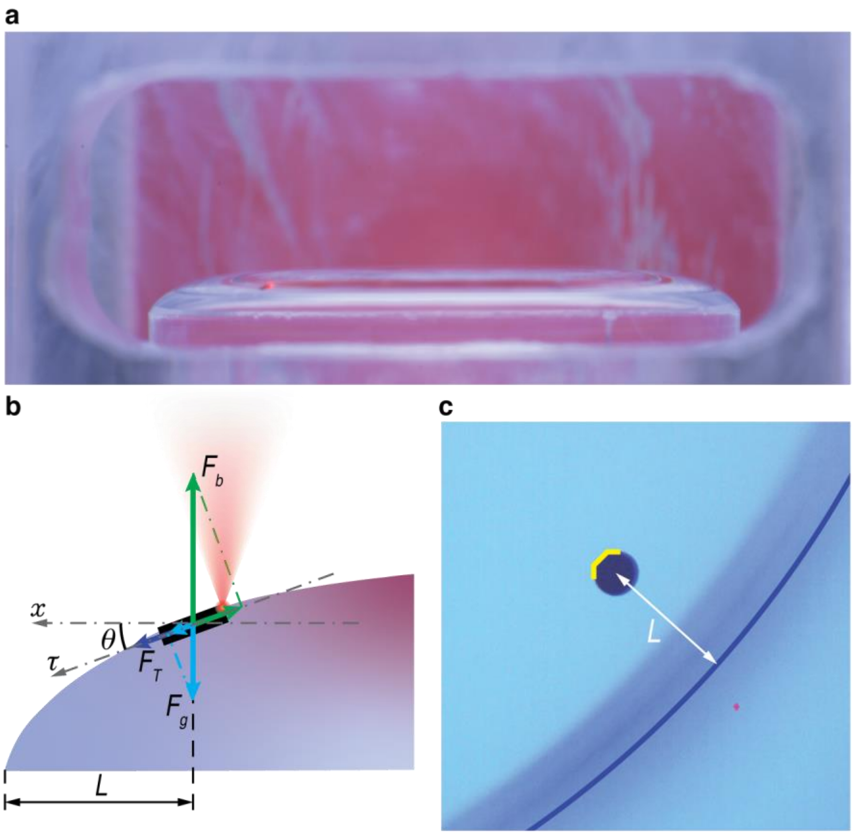

Figure S13. **Experimental measurement of the thermocapillarity-induced force. a)** Convex meniscus profile photo made through the side window of the chamber. **b)** Scheme and free-body diagram of the experiment. Axis x is perpendicular to gravity, axis τ is tangential to the liquid surface in the object's center. The particle (black rectangle) resides on the surface of the liquid at the distance L from the edge of the chamber; F_b (green) – buoyancy force; F_g (cyan) – gravitational force; F_T (blue) – thermocapillary force; **c)** Microscope view of the experiment, yellow line represents the laser trace, blue line shows the edge of the chamber.

References:

1. N. van Omme, L. Perron, V. Furnon, *or-tools user's manual*. *Rapp. tech. Google* (2014).
2. R.E. Kalman, A new approach to linear filtering and prediction problems. *J. Basic Eng.* **1960**, *82*, 35.
3. H. W. Kuhn, The Hungarian method for the assignment problem. *Nav. Res. Logist. Q.* **1955**, *2*, 83–97.
4. E. Jones, T. Oliphant, P. Peterson, others. SciPy: Open source scientific tools for Python.
5. S. Someya, T. Munakata, Measurement of the interface tension of immiscible liquids interface. *J. Cryst. Growth* **2005**, *275*, e343–e348.
6. B.A. Grzybowski, N. Bowden, F. Arias, H. Yang, G.M. Whitesides, Modeling of menisci and capillary forces from the millimeter to the micrometer size range. *J. Phys. Chem. B* **2001**, *105*, 404–412.
7. P.-G. De Gennes, F. Brochard-Wyart, D. Quéré, Surface tension. in *Capillarity and wetting phenomena: drops, bubbles, pearls, waves* 6–9 (Springer Science & Business Media, 2013).
8. R. Goldman, Curvature formulas for implicit curves and surfaces. *Comput. Aided Geom. Des.* **2005**, *22*, 632–658.

Captions for movies

Movie S1. Assembly and interconversion of colloidal lattices controlled by low-power light. This movie accompanies main-text **Figure 3**.

Movie S2. Parallelized, dynamic assembly of submillimeter parts controlled by low-power light. Letters in top-left corner correspond to subpanel labels from main-text **Figure 4**.

Movie S3. Assembly of a planetary gear system on the microstructured support (to accompany main-text **Figure 5a**).

Movie S4. Tethering of a 300 μm gear with 95 μm inner opening onto a 80 μm shaft (to accompany main-text **Figure 5d**).

Movie S5. Manipulation of droplets and droplet crystals by laser-controlled microbots. Letters in top-left corner correspond to subpanel labels from main-text **Figure 6**. Yellow trace is the laser trajectory; Magenta points represent object destinations; teal disks are GUI widgets used to specify microbot destinations.

Movie S6. Manipulation of non-absorptive objects (in order: 31 and 16 hollow glass microbeads, brass gear, and 3 white polyethylene beads) on FC-40 / DMF+Methyl Blue interface (to accompany main-text **Figure 7, c** to **e**). Left is the view from the software (microscopic), right is the view from DSLR camera. Yellow trace is the laser trajectory (and, in the case of brass gear, its contour); teal disks briefly reveal destination points of 31 glass bead in quasicrystalline lattice.

Movie S7. Transitions from square to hexagonal and from hexagonal to triangular patterns of green polyethylene microbeads driven by white light from a slide projector. Video of patterns persisting between the transitions was cut.
